# *Alpiniae oxyphyllae* Fructus in Alzheimer’s Disease: Multi-Target Mechanisms, Current Evidence, and Future AI-Driven Perspectives

**DOI:** 10.3390/molecules31203681

**Published:** 2026-10-14

**Authors:** Qihan Qin, Qingmin Yan, Keyi Zhu, Jingyuan Miao, Miaoxin Yang, Zhenghao Wu, Chenhan Fan, Ping Jiang, Guoqing Wu, Qinwen Wang

**Affiliations:** Health Science Center, Ningbo University, Ningbo 315211, China; 15923715363@163.com (Q.Q.); 13394614397@163.com (Q.Y.); 15990422511@163.com (K.Z.); 15372994391@163.com (J.M.); 18872908519@163.com (M.Y.); w13957825176@126.com (Z.W.); 18266972363@163.com (C.F.); 18252674943@163.com (P.J.)

**Keywords:** Alzheimer’s disease, *Alpiniae oxyphyllae* Fructus, extraction process, bioactive metabolites, artificial intelligence

## Abstract

*Alpiniae oxyphyllae* Fructus (Zingiberaceae; *Alpinia oxyphylla* Miq) (AOF) is a traditional Chinese medicinal and edible homologous material rich in bioactive metabolites, including flavonoids, terpenes, and sesquiterpenes. Its multiple metabolites and multi-target nature confer unique advantages for cognitive function and neuroprotection. However, recent advances in extraction techniques, the discovery of AOF effects, and novel ameliorative mechanisms against Alzheimer’s disease (AD) require an updated review. This review systematically expands on the botanical features, traditional dietary applications, and quality control of AOF raw materials, as well as its AD-ameliorating effect, in vivo absorption, toxicological safety, and extraction technology optimization strategies. Moreover, we explored the emerging mechanisms underlying the effectiveness of AOF and its bioactive metabolites in improving AD, including effects on glial cell function, the gut–brain axis, brain metal ions, and advanced glycation end products. This review comprehensively summarizes preclinical studies, clinical research, and commercially available products related to AOF and its metabolites. It also expounds upon the potential impetus that artificial intelligence could offer AOF research in the future. In summary, this review provides innovative technologies, in-depth mechanistic analyses, and novel research directions for exploring AOF extracts and their bioactive metabolites to offer strategic guidance for identifying new functional factors and drug candidates to combat AD.

## 1. Introduction

Traditional Chinese herbal medicines, characterized by multiple bioactive metabolites and multifaceted regulation, possess comprehensive ameliorative advantages [1]. Exploring new monomeric substances derived from Chinese herbal medicines is important for improving Alzheimer’s disease (AD). *Alpiniae oxyphyllae* Fructus (Zingiberaceae; *Alpinia oxyphylla* Miq) (AOF), a traditional Chinese medicinal herb [2], has a long history of medicinal use and has demonstrated remarkable potential to improve cognitive function and provide neuroprotection [3]. Studies have shown that various bioactive metabolites in AOF can exert pharmacological activities, including antioxidant, anti-inflammatory, and anti-amyloid deposition properties [4]. These effects reduce neuroinflammatory damage and inhibit neuronal degeneration, thereby providing new targets for improving AD.

The effects of AOF on AD have been summarized in previous studies [2,4,5,6,7]. However, recent studies indicate that glial cell dysfunction [8], gut–brain axis imbalances [9], disorders of brain metal ion homeostasis [10], and other mechanisms play non-negligible roles in AD. Moreover, systematic overviews regarding the preclinical and clinical development of AOF extract and its metabolites remain scarce, and comprehensive evaluations of commercially approved AOF-related products are still lacking.

Of particular importance, the emergence of ChatGPT (GPT-3.5) in 2022 initiated a new era of generative artificial intelligence (AI) [11]. Since then, large language models, represented by ChatGPT [12], Bard (Google) [13], LLaMA (Meta) [14], and DeepSeek (Zhi pu AI) [15], have undergone continuous iteration and optimization. These advancements have consistently enabled the application of deep generative models for predicting drug molecules and their functions, offering new possibilities for drug discovery, disease prevention, and control. AI, with its exceptional capabilities in data processing and deep analysis [16], holds great potential to optimize extraction processes [17], efficiently mine bioactive metabolites, accurately predict their functions, and explore AD targets using multi-omics and multimodal technologies. These research advancements warrant systematic summarization, and the future development trends of AOF in AD prevention and improvement require an in-depth discussion.

Therefore, based on recent scientific literature, this review systematically summarizes the emerging mechanisms through which AOF extracts and their bioactive metabolites exert ameliorative effects on AD. It also evaluates relevant clinical research findings and commercially approved products. In particular, it provides a comprehensive overview of prospective AI applications for future AOF research. This review aims to identify monomeric metabolites or functional factors in AOF that are effective at improving AD, thereby offering innovative insights for AD research.

## 2. AOF Raw Material, AOF Extract, and Extraction Technology

### 2.1. AOF Botany, Dietary Usage Habits, and Quality Control

**Figure 1:**
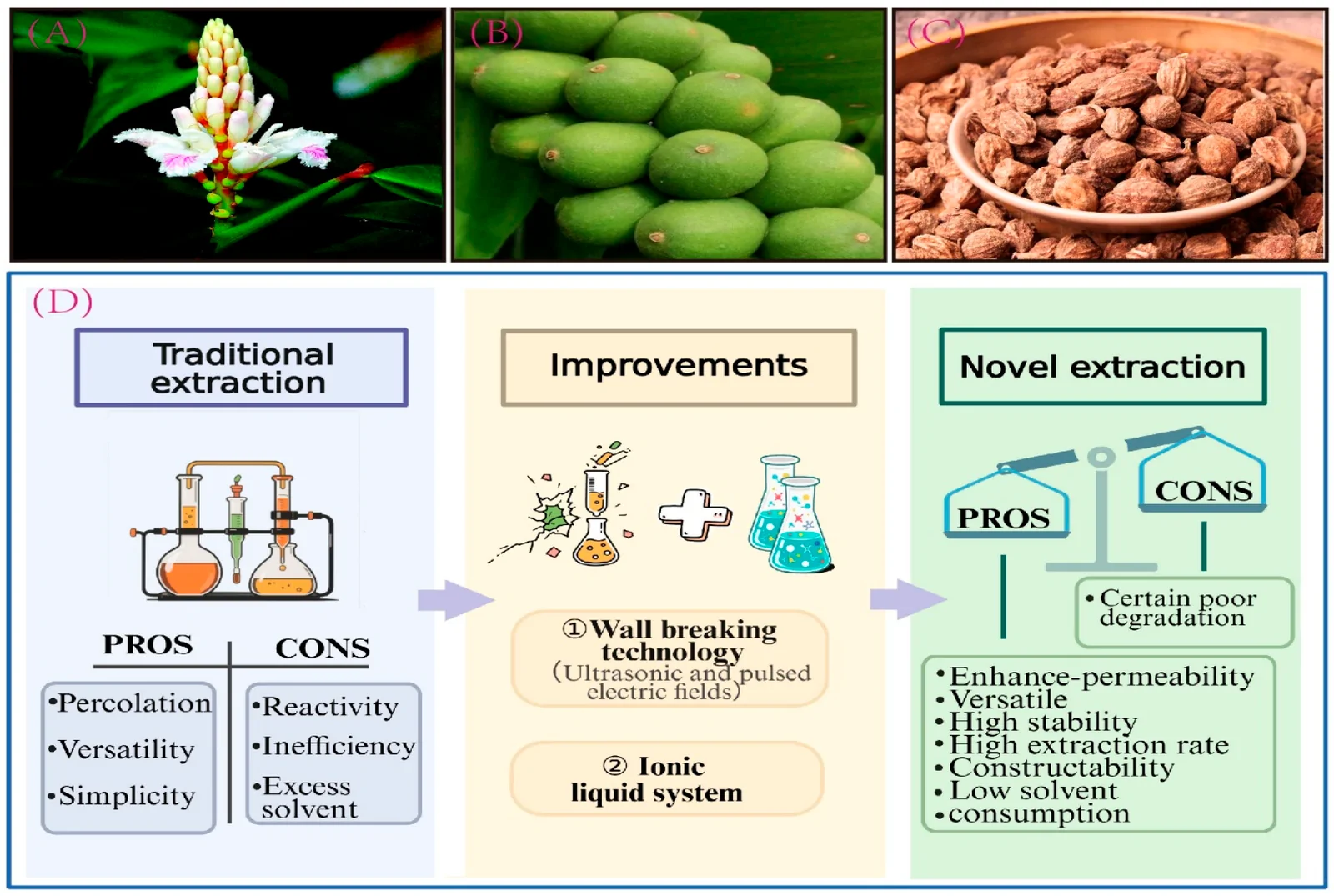
The botany of AOF and the technological innovation of novel AOF extraction process. (**A**–**C**) present the flower, fresh fruit, and dried fruit of AOF. (**D**) The technological innovation and advantages of the novel AOF extraction process.

AOF is a perennial herb of the genus *Alpinia* in the family *Zingiberaceae* and is recognized as one of the renowned “Four Southern Medicinal Herbs” in China. The morphology of its flowers and fruits is presented in Figure 1A,B. This plant prefers a warm and humid climate and is commonly found in shaded, moist environments under forest canopies. It is native to China and is primarily distributed in Hainan and Guangdong provinces. Its fruits are harvested when their color transitions from green to red and are subsequently air-dried in a well-ventilated, cool place or sun-dried to obtain the raw material for AOF (Figure 1C). Hainan province is a major raw-material-producing region for AOF. According to the Chinese Pharmacopoeia [18], the AOF fruit has a distinctive aroma and a pungent and slightly bitter taste. Its seeds have blunt edges and a diameter of about 3 mm; its surface is grayish yellow to grayish brown, with wavy wrinkles. AOF fruits are covered with a light brown membranous pseudo-endocarp and are hard in texture; the endosperm is white.

AOF possesses dual medicinal and edible properties and is classified as a non-toxic herb in the *Chinese Pharmacopoeia*, with a recommended daily dose of 3–10 g. Historically, AOF has been applied in traditional Chinese medicine and cuisine, with a usage history dating back 2000 years to the Han Dynasty in the Lingnan region. It was eaten fresh or processed by honeying, pickling, or soaking in wine. Today, it remains a popular local specialty in Hainan and Guangdong, China.

According to the Chinese Pharmacopoeia, the quality control of AOF raw material mainly includes the following aspects: microscopic identification (cross-section of the seeds), physicochemical identification (thin-layer chromatography of the powder), and determining the total ash content (not exceeding 8.5%), the acid-insoluble ash content (not exceeding 1.0%), and the microbial limit (compliant with relevant standards) [18]. Moreover, the active metabolite recorded in the Chinese Pharmacopoeia is volatile oil (no less than 1.0%) [18]. As research on AOF advances, an increasing number of active components have been identified, including flavonoids, terpenoids, and sesquiterpenoids. Meanwhile, recent advanced methods have enabled the more precise quantification of key compounds; such methods include chiral high-performance liquid chromatography–multiple reaction monitoring–mass spectrometry [19] for enantiomer-specific analysis and high-performance liquid chromatography–Fourier transform–ion cyclotron resonance–mass spectrometry [20] for multi-constituent profiling.

### 2.2. AD-Ameliorating Effect and the Absorption and Toxicity of AOF Extract

The extraction process for AOF varies with the target bioactive metabolites. The steam distillation method is most often adopted when volatile oil is the main extraction target. For the simultaneous enrichment of volatile oil, flavonoids, terpenoids, and other bioactive metabolites, ethanol and other organic solvents are commonly used for extraction. In one study, AOF extracts were separately prepared from the AOF raw materials (Tongren drug store, Shenyang, China) using ethanol, petroleum ether, chloroform, ethyl acetate, n-butanol, and water [21]. Based on comparisons with a control group, all AOF extracts (continuous intragastric administration for 14 days, 360 mg/kg) and donepezil (DPZ) (0.65 mg/kg) could significantly improve the cognitive function of lipopolysaccharide (LPS)-induced AD mice [21]. The AOF extract obtained via petroleum ether extraction showed the strongest effect, comparable to the positive control drug DPZ. The second-best effect was observed in the AOF extract via chloroform extraction. Moreover, compared to the other groups, he petroleum ether and chloroform groups could significantly inhibit the high expression levels of IBA-1, IL-1β, Aβ1–42, and phosphorylated tau protein (p-tau) in the hippocampus and cortex of mice [21]. This truly reflects the influence of different solvent systems on the effects of AOF extracts. Considering cost, safety, and other practical factors, ethanol extraction is the prevailing industrial approach for AOF large-scale production.

To clarify the in vivo process of AOF ethanol extract, its absorption, distribution, and metabolism characteristics were evaluated through pharmacokinetic analysis. It has been shown that following a single oral administration of AOF ethanol extract at a dosage of 54 mg/kg/d to male SD rats, the predominant bioactive metabolites reached maximum concentrations within the gastrointestinal tract, with the liver exhibiting the second-highest levels. Moreover, the tissue concentration–time curves for most tested tissues displayed a characteristic double-peak profile. Notably, metabolites such as nootkatone, yakuchinone A, and tectochrysin could be detected in brain tissue [22]. Another study indicated that 59 metabolites were identified in abdominal aorta blood samples from SD male rats (200–220 g) after a single oral administration of AOF ethanol extract at a dosage of 12.2 mg/kg/d (AOF raw material from Tongrentang Co., Ltd., Beijing, China) [23]. Furthermore, a total of 29 AOF-derived metabolites were identified in brain tissue samples, mainly diterpenoids, suggesting that these components have good potential to penetrate the blood–brain barrier [23].

AOF is widely recognized as a plant with both medicinal and edible properties. According to the Chinese Pharmacopoeia, the safe dosage range for the raw material of AOF is 3–10 g per person per day [18]. AOF extract is conventionally prepared using a 10:1 raw material-to-product mass ratio (10 kg of raw materials yields 1 kg of extract). Accordingly, the safe daily intake for an average 70 kg human subject ranges from 0.3 to 1.0 g. According to the body surface area method, the calculation results in an equivalent daily intake for mice of 39–130 mg/kg/d (with a conversion factor of 9.1) at a rate of 26.6–88.6 mg/kg/d (with a conversion factor of 6.2). Moreover, in an acute oral toxicity study, SD rats (male, weighing 200 ± 50 g, 10–12 weeks old) were administered a single intragastric high dose (1000 mg/kg) of crude AOF ethanol extract or AOF petroleum ether extract. The results showed that none of the rats exhibited any toxic or death symptoms over the 7 consecutive days of observation, preliminarily indicating that the AOF extract was well tolerated [24]. A study showed that 10-week-old male ICR mice (25–30 g) did not exhibit obvious toxic manifestations after oral administration of a high dose (360 mg/kg) of AOF extract with six different solvents derived from ethanol, petroleum ether, chloroform, ethyl acetate, n-butanol, and water solvents [21]. However, long-term safety, liver toxicity and other key toxicological indicators after long-term intervention with AOF extract remain unclear. Independent and systematic toxicological studies need to be carried out in the future to confirm these aspects.

### 2.3. AOF Extraction Process and Its Optimization Strategy

At present, the industrial-scale extraction of AOF primarily relies on solvent extraction methods, including aqueous and organic solvent extraction. The conventional solvent extraction method predominantly employs solvents such as methanol, ethanol, acetonitrile, propanol, or acetone for reflux extraction [25,26]. This method is characterized by strong solvent penetration, high equipment versatility, convenient operation, and controllable cost, making it a routine process in both laboratory research and industrial production. However, this method has notable limitations, including an unexpected chemical reaction, limited extraction efficiency, and excess solvent consumption. Furthermore, modern pharmacological research indicates that bioactive metabolites with neuroprotective properties exhibit significant diversity. Consequently, it is challenging to efficiently and simultaneously extract multiple types of bioactive metabolites using the conventional solvent extraction method.

In response to the aforementioned bottlenecks, recent efforts to optimize the extraction process have focused on two key strategies: wall-breaking technology and novel solvent systems. Wall-breaking physical methods, such as microwaves, ultrasound and pulsed electric fields, can improve cell wall permeability. For instance, the microwave extraction method efficiently extracts 25 bioactive metabolites from AOF within 8 min, including 7 novel terpenoid derivatives undetectable using conventional methods [27]. Furthermore, the ultrasonic-assisted method has been used to enhance the ethanol extraction process, resulting in improved yields of total flavonoids, terpenoids, and volatile oils from AOF [28].

In addition, remarkable advancements have been achieved in the exploration and development of novel solvent systems. As a novel type of solvent, ionic liquids are entirely composed of ions and exist in the liquid state under ambient conditions. Compared with traditional solvents, ionic liquids exhibit distinct advantages; they are colorless and odorless, possess excellent solubility and a broad dissolution range, and demonstrate high stability, a high extraction rate, and low solvent consumption. However, some ionic liquids exhibit poor biodegradability and are resistant to degradation, which may result in environmental pollution. Currently, deep eutectic solvents (DESs), comprising two or more natural, cost-effective, and biodegradable metabolites, represent a novel class of non-toxic, safe, efficient, and sustainable ionic liquid solvents. While retaining the advantages of traditional ionic liquids, DESs also exhibit high biodegradability, straightforward preparation, and biocompatibility. Owing to these properties, DESs have been gradually applied to the efficient extraction of bioactive components, including flavonoids, saponins, polysaccharides, alkaloids, phenolic acids, quinones, and volatile oils from plants and traditional Chinese medicines. For example, Nam et al. [29] employed customized DESs to extract flavonoids from Sophora flowers. In comparison to the conventional solvent, methanol, the extraction yield of total flavonoids was enhanced by 3%, while the free radical scavenging activity improved by 64%. However, DES applications in the extraction of bioactive components from AOF remain unexplored. Future studies need to investigate the feasibility of DES usage in the extraction of AOF and optimize solvent conditions to improve the extraction efficiency of target metabolites.

In conclusion, the advantages of the novel extraction process are evidenced by the synergistic integration of wall-breaking technology, and the new solvent system is shown in Figure 1D. Although the current application of DESs in the extraction of AOF is limited, their distinctive solvent characteristics suggest significant potential for future applications.

## 3. Bioactive Metabolites of AOF

### 3.1. Flavonoids

Flavonoid metabolites, a class of polyphenols, serve as key indicators of AOF quality. These compounds typically exhibit anti-inflammatory effects, decelerate oxidative damage, reduce acetylcholinesterase (AChE) activity, and inhibit Aβ accumulation. The primary bioactive flavonoid components detected in AOF include chrysin, daucosterol palmitate, tectochrysin, kaempferol, tectochrysin A, kaempferol-7, 4′-dimethylether, izalpinin, myricetin, baicalein, pinocembrin, rhamnocitrin, and galangin. Among these, chrysin [30], daucosterol palmitate [31], tectochrysin [32], kaempferol [33], myricetin [34], baicalein [35], and pinocembrin [36] have been shown to exert AD-ameliorating effects. Moreover, other flavonoids in AOF have been identified and validated in animal experiments. For instance, quercetin ameliorates AD in animal models [37], yet it occurs at a relatively low level in AOF. Additionally, studies on AD cell models have confirmed the neuroprotective effects of izalpinin [38]. Thus, the neuroprotection conferred by flavonoids in AOF is achieved through multi-target regulatory mechanisms with low toxicological profiles.

### 3.2. Terpenoids

Terpenoids, including monoterpenes, sesquiterpenes, diterpenes, and triterpenoids, are the most abundant active components of AOF. Diterpenoids and triterpenoids are present in AOF at extremely low levels and are difficult to separate, with most studies only achieving chemical identification. In AOF, eucalyptol, linalool, α-terpineol, β-pinene, β-sitosterol, limonene, and D-limonene are relatively common terpenoid metabolites. Novel bioactive metabolites, including alpinoxyphyllols A, alpinoxyphyllols B, 2-O-ethyl-β-nootkatol, 11-hydroxyisohanalpinone, 6S-/6R-oxyphyllenone H [39], and cyperusol A4 [40], have been identified. However, only a limited number of metabolites have been verified to exert clear neuroprotective and AD-ameliorating effects in animal models. These include eucalyptol [41], linalool [42], α-terpineol [43], β-pinene [44], and β-sitosterol [45]. Furthermore, although studies on limonene [46] and D-limonene [47] in AD models have not yet been conducted, these two compounds have exhibited neuroprotective effects in a Parkinson’s model and aluminum chloride-induced dementia model, respectively.

Among terpenoids, only 2β-hydroxy-δ-cadinol [48] has been investigated in AD cell studies. Most of the remaining terpenoids have only been identified, with no experimental reports on their effects in terms of improving AD; further studies on their pharmacological activities and mechanisms are warranted. Moreover, although cell experiments allow precise variable control, they lack the holistic regulation of living organisms. Accordingly, in vitro findings may not fully reflect the actual pharmacological effects observed in vivo.

### 3.3. Sesquiterpenoids

Sesquiterpenoids, a class of terpenoid metabolites, are crucial in AOF and possess multiple neuroprotective effects, such as anti-inflammatory, antioxidant, anti-apoptotic, and anti-AChE activities. In previous animal studies exploring the sesquiterpenoids of AOF, metabolites such as nootkatone [49] and β-caryophyllene [50] were investigated for their in vivo effects in ameliorating AD. Moreover, the antioxidant and anti-inflammatory properties of valencene have been confirmed in other animal models, but their role in the AD animal model has not been reported yet [51].

In terms of cell experiments, the anti-neurotoxic activity of 9-hydroxy epinootkatol [52] and the anti-inflammatory effects of (5R,7S,10S)-E-eudesma-4(15),11-dien-7-ol, teuhetenone, litseagermacrane, and 1-oxo-5α,7αH-eudesm-3-en-15-al [53] have also been confirmed in non-AD cell models. To date, these metabolites have not yet been investigated in AD cell models. However, these experiments were conducted in vitro; therefore, the actual ameliorative potential and mechanisms of action of these sesquiterpenoids remain unclear. Further studies on animal models are required to evaluate their pharmacological activity, pharmacokinetics, and ameliorative effects.

Furthermore, in thin layer chromatography and bioactivity detection experiments, (11S)-12-chloronolone-11-ol, (11R)-12-chloronolone-11-ol, 12-noracyl-6-ene-11-one, oxyefolone, naphthoquinone, 9β-hydroxynaphthosterone, oxyefolene A, (9E)-humulen-2,3; 6, 7-dioxide, alpine ketone, and pubescone have been shown to possess anti-AChE activity [54]. However, these studies remain limited to qualitative in vitro activity screening. Some of these metabolites may be pan-assay interference compounds (PAINS), which are chemical compounds that frequently yield false positive results in high-throughput screening experiments [55]. Consequently, due to the lack of quantitative effect evaluations and in vivo experimental validation, the true ameliorative potential and mode of action of these compounds remain unverified, warranting further exploration before they can be considered viable drug candidates.

### 3.4. Volatile Oils

Volatile oils are a mixture of aromatic and volatile metabolites extracted from the seeds and fruits of AOF. Valencene, nootkatone, and p-cymene are the main components in volatile oil [2]. The pharmacological effects of valerene and nootkatone in animals are presented in detail in Section 3.3 “Sesquiterpenoids”. Moreover, the AD-ameliorating effects of p-cymene have been reported in rats [56]. Furthermore, although these metabolites, including α-pinene [57], myrtenal [58], camphene [59], citronellol [60], β-elemene [61], sabinene [62], and carveol [63], occur at relatively low levels in AOF volatile oil, animal experiments have validated their neuroprotective effects, but relevant investigations regarding AD have not yet been reported. In summary, essential volatile oils obtained via hydrodistillation were analyzed using gas chromatography mass spectrometry, identifying 74 constituents; apart from the aforementioned substances, the majority of them have yet to be associated with AD or neuroprotective effects [64].

### 3.5. Diarylheptanoids

Diarylheptanoids are characteristic chemical components of AOF. Yakuchinone A, oxyphyllacinol, and yakuchinone B are the primary metabolites [65]. The other seven metabolites have been isolated from diarylheptanoids of AOF, including neonootkatol, dihydrogingerenone B [66], 1-(3′,5′-dihydroxy-4′-methoxyphenyl)-7-phenyl-3-heptanone [67], 1-(2′,4′-dihydroxy-3′-methoxyphenyl)-7-(4′-methoxyphenyl)-3-heptanone [67], 1,5-epoxy-3-hydroxy-1-(4-hydroxy-3,5-dimethoxyphenyl)-7-(4-hydroxy-3-methoxyphenyl)-heptane [68], (E)-1-(4′-hydroxy-3′-methoxyphenyl)-7-(4″-hydroxyphenyl)-hept-4-en-3-one [69], and yakuchinester A [38]. However, they remain unreported in AD animal studies; only yakuchinone A [38] and yakuchinester A [38] have been studied in AD cells. Neuropharmacological research on most of these compounds remains in its early stages, as most available evidence has been derived from in vitro chemical assays. For instance, in 1,1-diphenyl-2-picrylhydrazyl free-radical-scavenging assays to evaluate antioxidant capacity, several diarylheptanoids, including 1-3′, 5′-dihydroxy-4′-methoxyphenyl)-7-phenyl-3-heptanone and 1-(2′,4′-dihydroxy-3′-methoxyphenyl)-7-(4″-methoxyphenyl)-3-heptanone, have demonstrated potent antioxidant activities [67]. To mitigate PAINS-related interference, future studies should prioritize independent in vivo valuations of purified monomers, such as oxyphyllacinol, yakuchinone A, and yakuchinone B, in established AD models to clarify their pharmacokinetic profiles and the actual contributions to cognitive improvement.

### 3.6. Other Ingredients

In addition to flavonoids, terpenoids, sesquiterpenoids, diarylheptanoids and volatile oils, AOF also contains neuropharmacological bioactive metabolites such as phenolic acids, furans, and methylxanthines. To date, studies concerning these compounds remain limited, so future, more comprehensive investigations are warranted. 

In conclusion, this review summarizes studies demonstrating the AD-ameliorating effects of various active metabolites in AOF in animal models (Table 1). Studies reporting bioactivity only in cell experiments are compiled in Table 2. AOF has significant research value due to its diverse composition, which includes flavonoids, terpenoids, sesquiterpenoids, diarylheptanes, volatile oils, and other bioactive metabolites. Collectively, these compounds contribute to the pharmacological activity of AOF. Specifically, studies have shown that AOF can block the generation of β-amyloid protein (Aβ) and abnormal p-tau, thereby exerting antioxidant and anti-inflammatory effects, promoting neurogenesis, and inhibiting apoptosis [4]. These properties offer promising strategies for investigating and improving AD.

## 4. AOF and Metabolites: Multi-Target AD

Network pharmacology and molecular docking analyses predicted that AOF may modulate neurodegenerative processes through multi-target mechanisms, including Aβ deposition, tau protein metabolism, and neuroinflammation [70,71]. Network analysis aims to identify potential drug targets and generate mechanistic hypotheses; however, its application in ethnopharmacology is constrained by limited predictive validity and insufficient biological relevance due to multi-layered homogeneity across distinct research objects [72]. Considering the inherent methodological limitations of network analysis outlined above, this review systematically integrates solid evidence from wet experiments including in vivo animal studies and in vitro cellular assays, and such empirical data (especially in in vivo experiments) serve as more authoritative and reliable references for mechanistic exploration and pharmaceutical development.

### 4.1. Aβ and P-Tau

It is well known that the abnormal deposition of Aβ and hyperphosphorylation of the tau protein, resulting in the formation of neurofibrillary tangles, are classical pathological features of AD. They jointly induce neuronal loss, synaptic dysfunction, and progressive cognitive decline. AOF extracts (petroleum ether or ethanol extract; Tongren drug store, Shenyang, China) were administered to mice by gavage at a dose of 360 mg/kg for 14 days. These improvements significantly reduced Aβ deposition and reversed hippocampal p-tau levels in mice with LPS-induced AD [21]. In contrast, under similar experimental conditions, an n-butanol extract of AOF (Tongrentang Drug Co., Ltd., Shenyang, China) exhibited dose-dependent effects over a 20-day improvement period. A dose of 360 mg/kg significantly reduced Aβ deposition in mice with Aβ-induced AD, whereas a dose of 180 mg/kg was ineffective [73].

Moreover, many bioactive metabolites in AOF have been shown to reduce Aβ levels in mouse models of AD. In the presence of DPZ (3 mg/kg) as a positive control, continuous oral administration of protocatechuic acid at 100 mg/kg for 4 weeks significantly reduced Aβ deposition in the brains of APP/PS1 mice [74]. In an intravenous administration experiment, continuous administration of nootkatone (extracted from AOF; purity > 98%) for 5 days prevented Aβ deposition in the hippocampus of an Aβ-induced mouse model of AD, with a minimum effective dose of 0.02 mg/kg [75]. Using memantine (20 mg/kg) as a positive control, oral administration of baicalein (Chemical Centre, purity 98%) at 10 mg/kg for 21 consecutive days effectively reduced Aβ deposition in the hippocampi and cortices of rats with Aβ-induced AD [35]. Furthermore, a 3-month intervention with chrysin (MedChemExpress LLC., purity unspecified) at 25 mg/kg inhibited the formation of Aβ plaques in the hippocampi and cortices of APP/PS1 mice [30]. Similarly, tectochrysin administration (Jurong Pharmaceutical Co., purity > 99%) at doses of 14 or 140 μg/kg significantly inhibited the Aβ-induced increase in hippocampal Aβ accumulation in mice with AD [32]. In addition, in vitro experiments have shown that the administration of protocatechuic acid (Sigma, purity unspecified) at concentrations of 1, 5, and 50 μM, or combined pretreatment with nootkatone (2.5, 5, 10, and 20 μM) and schisandrin (12.5, 25, 50, and 100 μM) (source and purity unspecified), protected PC12 cells from Aβ-induced toxicity [76] and reduced p-tau and Aβ levels [77,78].

### 4.2. AChE

Cholinergic system dysfunction has been observed in various neurodegenerative diseases that affect cognition, memory, and emotion [79]. The cholinergic system relies on acetylcholine as a primary neurotransmitter, and AChE is the principal enzyme responsible for its degradation [80]. In AD, AChE expression levels increase due to neuronal degeneration, leading to increased activity [81]. Under negative control conditions, while using DPZ (0.65 mg/kg) as a positive control, continuous intragastric administration of the chloroform extract of AOF (Tongrentang Drug Co., Ltd., Shenyang, China) at 180 mg/kg for 20 days exhibited AChE inhibitory activity in Aβ-induced mice with AD [82].

Furthermore, many bioactive metabolites of AOF have demonstrated significant AChE inhibitory activity. For instance, nootkatone [75], chrysin [83], β-sitosterol [84], β-asarone [85], pinostrobin [86], and protocatechuic acid [87] can significantly increase the expression levels of AChE in the brain and alleviate the pathological symptoms of AD in mouse models. Specifically, intravenous injections of 0.02 and 0.2 mg/kg nootkatone (extract from AOF, purity > 98%) administered for 5 consecutive days inhibited AChE and partially mediated the neuroprotective effect against Aβ-induced cognitive impairment in mice [75]. In an experiment with DPZ (1.5 mg/kg) as the positive control, the oral administration of chrysin nanoparticles (Otto Kemi, purity unspecified) at a dose of 50 mg/kg for 4 consecutive weeks reduced AChE activity in the brains of scopolamine-induced rat models with neuronal injury [83]. Continuous oral administration of β-sitosterol (source and purity unspecified) at 25 mg/kg for 21 days significantly inhibited AChE activity in the hippocampi of mice with aluminum chloride-induced memory impairment, thereby increasing acetylcholine levels [84]. Furthermore, in a model where DPZ (5 mg/kg) served as a positive control, oral administration of pinostrobin (extract from fresh rhizomes of *Boesenbergia rotunda* (L.) Mansf. (*Zingiberaceae*; *Boesenbergia rotunda* rhizome), purity unspecified) for 14 consecutive days at doses of 20 or 40 mg/kg inhibited AChE activity, regulated cholinergic system function, and significantly reduced scopolamine-induced memory impairment [86].

Additionally, the anti-AChE activity exerted by limonene, baicalein, and myristin was confirmed using spectrophotometry, Ellman’s reagent method, and fluorescence determination [88,89,90,91]. However, due to the lack of quantitative effect evaluation and in vivo experimental validation, the possibility of PAINS-related interference cannot be excluded for these metabolites.

### 4.3. Glial Cells

**Figure 2:**
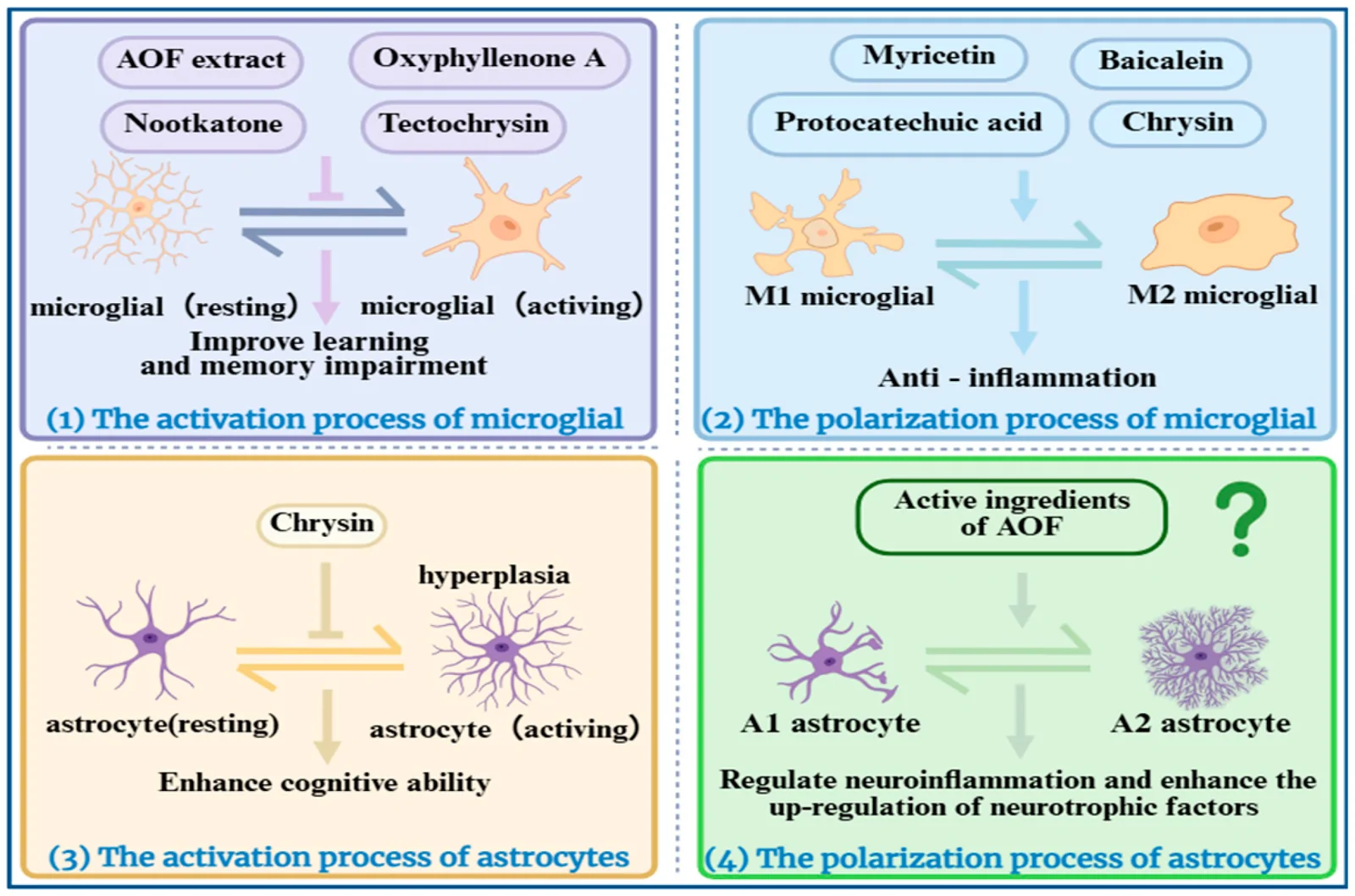
AOF extract and its bioactive metabolites improve the functions and homeostasis of microglia and astrocytes in the AD model.

Glial cells, particularly microglia and astrocytes, are indispensable for the development of AD. During the acute response phase of AD, the activation of microglia and astrocytes can prevent its progression through the release of anti-inflammatory cytokines that mediate Aβ clearance and prevent excessive synaptic phagocytosis. However, in the late stage of AD, excessive or abnormal microglial and astrocyte activation may further drive Aβ aggregation [92]. The bioactive metabolites of an AOF extract improved the function and homeostasis of microglia and astrocytes in mouse models of AD (Figure 2). (1) Activation of microglia: A 14-day intervention with petroleum ether or chloroform extracts of AOF (Tongren drug store, Shenyang, China) at 360 mg/kg significantly inhibited excessive microglial activation in the hippocampus and cerebral cortex and improved cognitive function in a mouse model of AD [21]. Similarly, oral administration of nootkatone (Dalian Meilun Biology and Technology Co. Ltd., Dalian, China, purity > 97%) at 5 mg/kg for 19 days suppressed hippocampal and cortical microglial hyperactivation, thereby ameliorating cognitive dysfunction in mice with LPS-induced AD [49]. Furthermore, oxyphyllenone A (MeilunBio, purity ≥ 98.0%) at concentrations of 3.125, 6.25, 12.5, 25, 50, and 100 μM; tectochrysin (MeilunBio, purity ≥ 98.0%) at concentrations of 1.5625, 3.125, 6.25, 12.5, 25, and 50 μM; and nootkatone (MeilunBio, Dalian, China, purity ≥ 98.0%) at concentrations of 1.5625, 3.125, 6.25, 12.5, 25, and 50 μM effectively inhibited the LPS-induced polarization of BV2 microglial cells toward the classically activated (M1) phenotype [93]. (2) Polarization of microglia: Intraperitoneal injection of myricetin (McLean Company, purity ≥ 97%) at 20 mg/kg for 3 consecutive weeks promoted the transformation of cortical microglia from the pro-inflammatory M1 phenotype to an anti-inflammatory alternatively activated (M2) phenotype in 3 × Tg-AD mice [94]. Continuous intragastric injection of baicalein (source and purity unspecified) at 100 mg/kg for 7 days promoted the polarization of microglial cells toward the anti-inflammatory M2 phenotype, thereby alleviating neuronal damage and inflammation in APP/PS1 mice [95]. Similarly, continuous intragastric administration of protocatechuic acid (Aladdin Chemical Co., Shanghai, China; purity ≥ 97%) at 30 mg/kg for 3 days promoted the transformation of mouse microglia from the pro-inflammatory M1 phenotype to the anti-inflammatory M2 phenotype following cerebral hemorrhage [96]. Chrysin (MeilunBio, purity ≥ 98.0%) at concentrations of 3.125, 12.5, 25, and 50 μM promoted anti-inflammatory M2 polarization in LPS-treated BV2 cells [93]. (3) Activation of astrocytes: Intragastrical administration of chrysin (Sigma, purity ≥ 96.5%), at a dose of 1100 mg/kg for 7 days, improved memory in rats with AD by inhibiting astrocyte activation [97]. (4) Polarization of astrocytes: Polarization of astrocytes toward the neuroprotective/anti-inflammatory reactive astrocyte phenotype can regulate neuroinflammation and upregulate neurotrophic factor levels, thereby exerting neuroprotective effects [98,99]. However, to date, no studies have elucidated the roles of AOF and its bioactive metabolites in astrocyte polarization.

### 4.4. Microbial Conduction Axis

**Figure 3:**
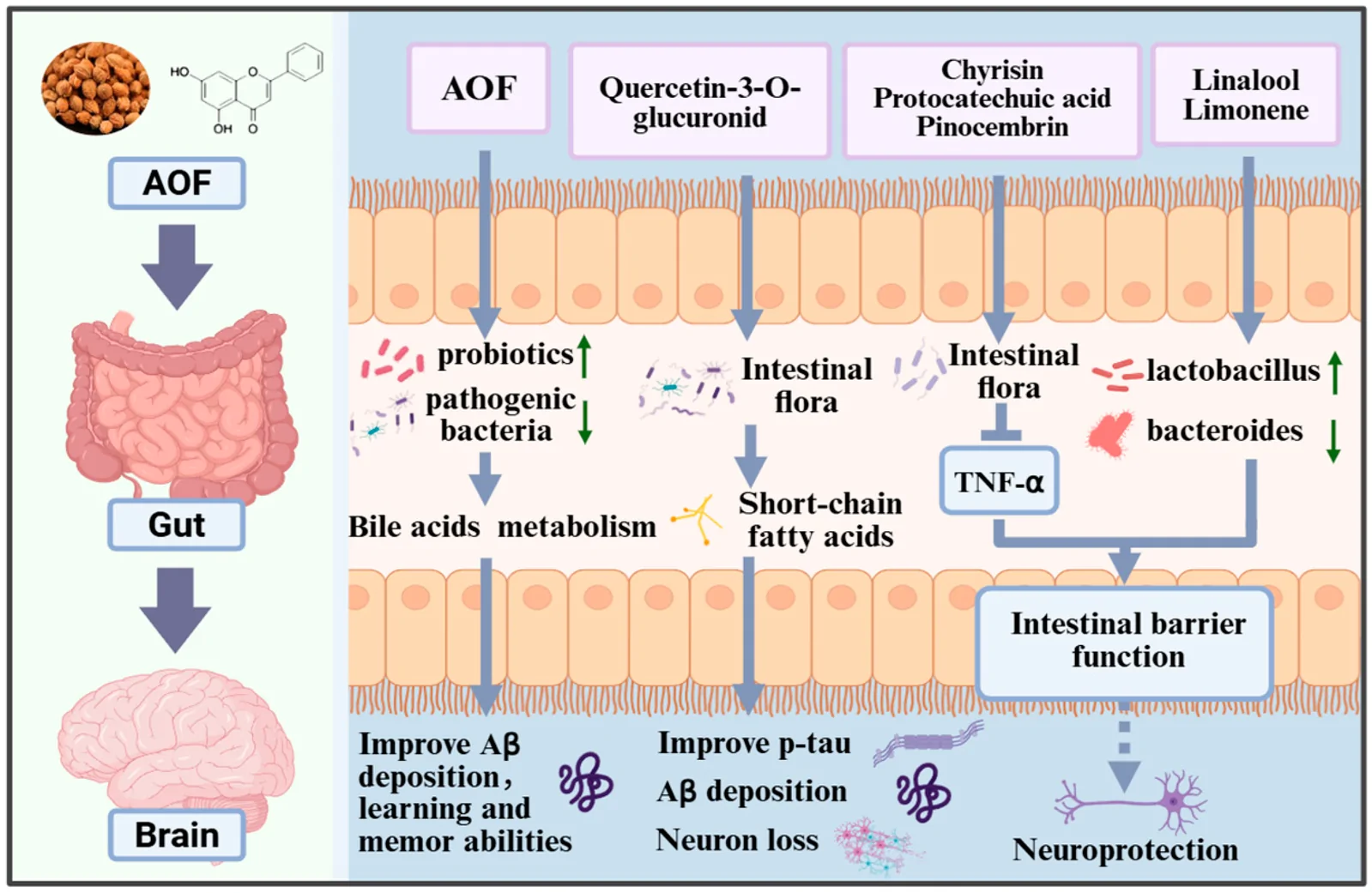
The effects of the extract of AOF and its bioactive metabolites on regulating AD based on the “microbiota–gut–brain” axis.

The mechanism by which the bioactive metabolites of an AOF extract modulate the microbiota–gut–brain axis to alleviate AD pathology is illustrated in Figure 3. Specifically, (1) the oral administration of an AOF extract (Tongrentang Drug Co., Ltd., Henan, China) at a dose of 0.5 g/kg for 3 consecutive months resulted in the regulation of intestinal probiotics and inhibition of pathogenic bacteria, alongside a decrease in the ratios of serum deoxycholic acid to cholic acid and lithocholic acid to chenodeoxycholic acid. These mechanisms likely rely on intestinal bile acid metabolism to facilitate regulation from the gut to the brain. This process may, consequently, increase Aβ deposition and enhance cognitive function in APP/PS1 mice [100]. (2) Furthermore, oral administration of 50 mg/kg quercetin-3-O-glucuronide (Sigma, purity unspecified) for 28 days decreased p-tau accumulation, Aβ deposition, and neuronal loss in mice with Aβ-induced AD by regulating gut microbiota dysbiosis and enhancing short-chain fatty acid production [101]. (3) Using 10 mg/kg atorvastatin as a positive control, continuous oral administration of 100 mg/kg chrysin (source and purity unspecified) for 30 days reversed the intestinal flora imbalance in mice with cerebral hemorrhage [102]. Similarly, using sulfasalazine (100 mg/kg) as a positive control, continuous oral administration of protocatechuic acid (MACLIN, purity > 98%) at doses of 5, 10, and 20 mg/kg for 7 days regulated the intestinal microbiota composition and inhibited the release of pro-inflammatory factors in mouse models of ulcerative colitis. Furthermore, continuous oral administration of protocatechuic acid (source and purity unspecified) at doses of 5 or 25 mg/kg for 15 days also inhibited the release of pro-inflammatory factors in a mouse model of inflammatory bowel disease [103,104]. Additionally, using 5-aminosalicylic acid (50 mg/kg) as a positive control, continuous oral administration of pinocembrin (Biopurify Phytochemicals Ltd., Chengdu, China; purity > 99%) at doses of 5 or 10 mg/kg for 14 days restored intestinal barrier function and regulated intestinal microbiota composition in a colitis model, particularly at the 5 mg/kg dose [105]. These intestinal changes may contribute to neuroprotective effects; however, direct evidence supporting regulation of the gut–brain–axis remains lacking. (4) In another study conducted without a control group, mice were orally administered 0.2 mL of an extract containing linalool and limonene from orange essential oil (Qu Gu Food Co. Ltd., Zigui, China) for 28 consecutive days. Linalool and limonene reduced the abundance of Bacteroides and increased the relative abundance of *Lactobacillus* in the intestines of normal mice, thereby restoring intestinal barrier function [106]. However, direct evidence linking these changes to neuroprotective effects is yet to be established.

### 4.5. Autophagy

**Figure 4:**
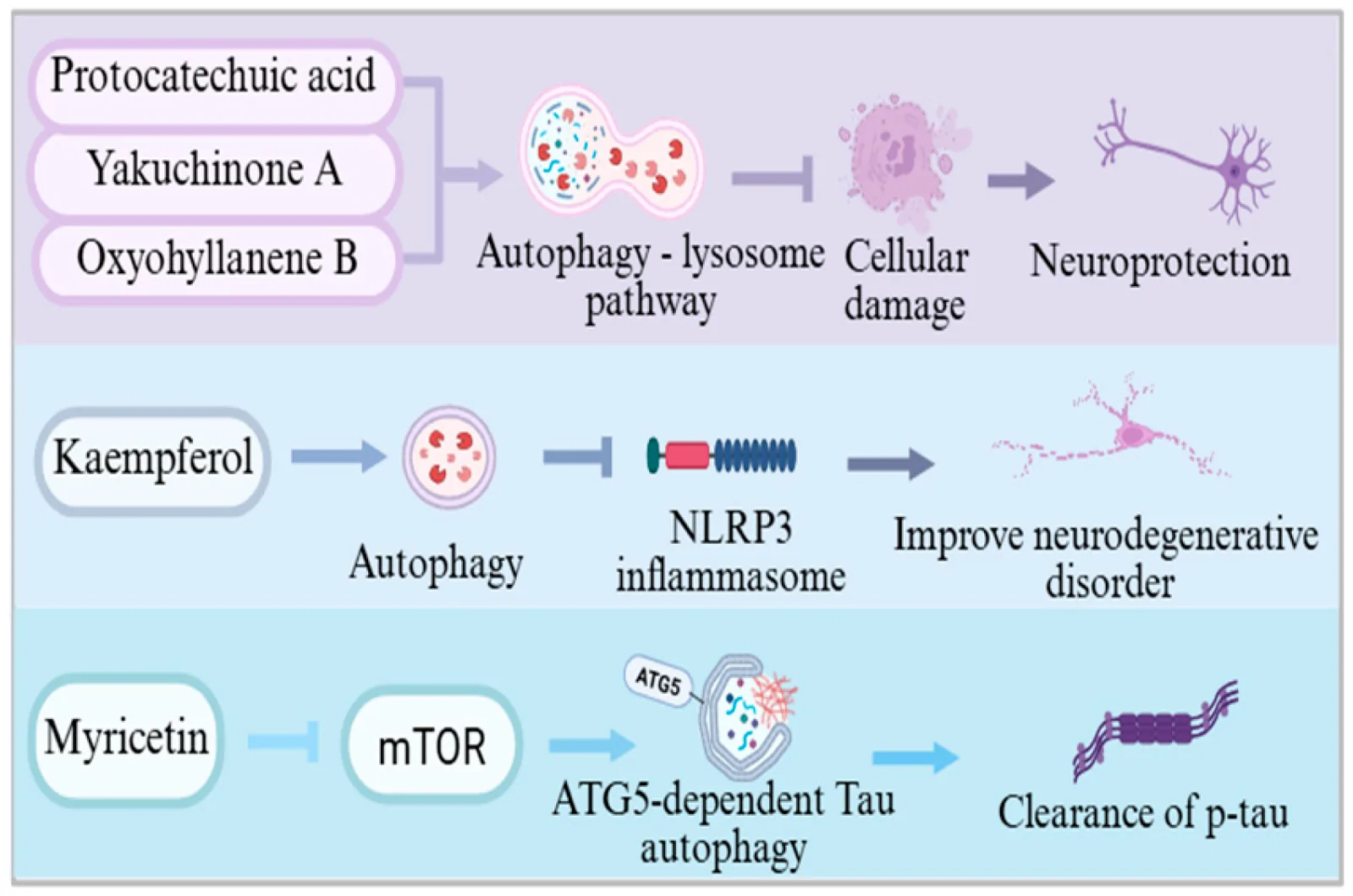
AOF bioactive metabolites in AD improvement via autophagy regulation.

Autophagy in the brain helps maintain neuronal function by removing abnormal proteins and damaged organelles and supporting the energy supply and metabolic balance of cells. In the early stage of AD, autophagy can clear the abnormal accumulation of Aβ and tau proteins and reduce neuronal damage. However, their continuous accumulation [107] induces abnormal autophagy, which worsens AD symptoms [108]. Bioactive metabolites in AOF can improve AD by regulating autophagy (Figure 4). Specifically, (1) protocatechuic acid (Sigma, purity unspecified, 400 mol/L) [109], as well as yakuchinone A and oxyphyllanene B (0.5 mM; extracted from AOF purchased from the traditional Chinese medicine market in Kunming; purity unknown) [38], activated the lysosomal autophagy pathway in primary hippocampal neurons and BV2 cells, increased autophagic activity, reduced cellular damage, and ultimately exerted neuroprotective effects. (2) Kaempferol administration (source and purity unspecified) at 40 μM significantly reduced activation of the NOD-like receptor family pyrin domain-containing protein 3 inflammasome in the cytoplasm of BV2 cells by promoting autophagy, thereby alleviating neurodegeneration. These findings suggest that kaempferol may counteract neuroinflammation through autophagy regulation [110]. (3) Myricetin (source and purity unknown, 10 μM) activated autophagy-related gene 5-dependent tau autophagy by inhibiting the mechanistic target of the rapamycin (mTOR) pathway, promoted p-tau clearance in SH-SY5Y cells, and almost completely inhibited tau-associated neurotoxicity [111]. It is worth noting that most current evidence regarding autophagy regulation is derived from in vitro cellular studies and lacks independent in vivo validation, pathway-specific blocking experiments, and genetic verification. Therefore, further studies are required to clarify the specific mechanisms by which natural compounds regulate autophagy and exert neuroprotective effects in vivo.

### 4.6. Metal Ions

Research has indicated that the accumulation of copper, iron, and zinc in brains with AD is 5.7, 2.8, and 3.1 fold higher than that in the normal brain, respectively [112]. Elevated metal ions can induce or exacerbate excessive Aβ production, tau hyperphosphorylation, and Aβ/tau aggregation, as well as activate oxidative stress, impair autophagy, and induce synaptic dysfunction [113]. For instance, intragastric administration of 50 mg/kg chrysin (source and purity unspecified) for 5 consecutive days significantly reduced the levels of Cu^2+^, Zn^2+^, and Fe^2+^ in the hippocampi of rats (21.6 mg/kg ginaton was used as a positive control) [114]. In addition, using 150 mg/kg of deferiprone as a positive control, the administration of baicalein (prepared by the Institute of Materia Medica, Chinese Academy of Medical Sciences & Peking Union Medical College, purity > 98%) at doses of 150 or 300 mg/kg for 3 weeks significantly reduced iron accumulation in the substantia nigra and inhibited iron-induced oxidative stress in rat brains [115]. Similarly, intragastric administration of myricetin (source and purity unspecified) at doses of 25 or 50 mg/kg for 6 consecutive days reduced iron content in the mouse brain and alleviated scopolamine-induced cognitive impairment in mice [116]. Furthermore, the administration of 50 μM myricetin (source and purity unspecified) regulated Aβ oligomerization and neuronal toxicity induced by Cu^2+^ and Zn^2+^ in SK-N-BE (2)-M17 cells [117]. Linalool (Merck Life Sciences Kft., Budapest, Hungary, purity unspecified, 0.54 μg/mL) altered the expression of iron-related genes and affected intracellular iron content in SH-SY5Y cells treated with 6-hydroxydopamine hydrobromide [118].

### 4.7. Exosomes

Exosomes transport disease-associated proteins in brains with AD, promote Aβ aggregation, and induce astrocyte apoptosis [119]. Thus, inhibiting exosome formation, secretion, and uptake has been shown to effectively reduce the transport of oligomers and their associated toxicities [120]. For instance, chrysin (Sigma, purity unspecified, 1 μM) increased the expression level of kruppel-like factor 2, the target gene of exosomal *microRNA-92a*, in human coronary artery endothelial cells and significantly reduced the level of exosomal *microRNA-92a* in a time-dependent manner [121]. Additionally, intraperitoneal injection of kaempferol inhibited exosome secretion by alveolar epithelial cells induced by sepsis, blocked exosome-mediated activation of the mitogen-activated protein kinase signaling pathway in alveolar macrophages, and ultimately alleviated sepsis-related acute lung injury [122]; however, the effect of kaempferol on AD via exosomes remains unknown. Notably, exosomes possess the inherent ability to cross the blood–brain barrier and retain their functional properties after entering the brain. This ability makes exosomes ideal carriers for drug delivery [123], which can enhance the bioavailability of drugs or active substances, facilitate brain targeting, and amplify their neuroprotective effects [124]. Hence, a combination of AOF bioactive metabolites and exosomes may effectively enhance the concentration and effects of AOF substances in the brain. However, reports on the neuroprotective effects of AOF and its exosomal metabolites remain limited.

### 4.8. Epigenetic Inheritance

More than 20 epigenetic mechanisms have been identified to be associated with AD. They primarily involve direct alterations of DNA (e.g., methylation), modifications of chromatin structure (e.g., histone modification), and adjustments to mRNA-related processes (including those involving ncRNAs and miRNAs) [125]. Many AOF metabolites, such as myricetin, kaempferol, chrysin, and baicalein, are effective against AD and improve brain health through classical epigenetic mechanisms. Intraperitoneal injection of myricetin (Sigma, purity unspecified) at doses of 50 mg/kg (minimum effective dose) or 100 mg/kg for 2 weeks alleviated sevoflurane-induced cognitive dysfunction in aged mice by downregulating histone deacetylase 2 expression and inhibiting nuclear factor erythroid 2-related factor 2 deacetylation in hippocampal neurons [126]. In an experimental autoimmune encephalomyelitis model, mice intraperitoneally injected with kaempferol (Sigma, purity ≥ 90%) for 32 days exhibited increased expression levels of *hsa-microRNA-367-3p* in the brain and spinal cord. Kaempferol may promote intracellular iron efflux by upregulating the expression of the iron transporter ferroportin 1, thereby inhibiting ferroptosis and exerting neuroprotective effects [127]. Oral administration of chrysin (Thermo Fisher, Waltham, MA, USA, purity unspecified) at 10 mg/kg to a rat model of autism for 22 consecutive days resulted in the regulation of histone deacetylase activity and the acetylation modification status in the cortexes and hippocampi. In addition, it promoted the synthesis of the key synaptic protein SH3 and multiple ankyrin repeat domains 3, and thereby alleviated behavioral deficits in autistic rats [128]. In the SD rat model of status epilepticus induced by lithium chloride and anisodamine, the administration of baicalein (source, purity, and intervention duration unspecified) at doses of 50 or 100 mg/kg) demonstrated dose-dependent antiepileptic and neuroprotective effects, with 50 mg/kg identified as the minimum effective dose. Additionally, mechanistic studies have shown that baicalein inhibited DNA methyltransferase 1 and reduced methylation of the promoter region of the γ-aminobutyric acid type A receptor subunit delta, thereby upregulating γ-aminobutyric acid type A receptor subunit delta expression. These effects helped restore inhibitory neurotransmission, rebalance the excitation–inhibition ratio in neural networks, and alleviate epilepsy-associated neuronal damage [129].

Previous research on AOF metabolites has predominantly focused on protein glycation, revealing that the glycation patterns of various key proteins change during the pathological progression of AD [130]. In a rat model of AD without a positive control group, gavage administration of pinocembrin (synthesized by the Department of Medical Synthetic Chemistry; purity > 98%) at doses of 20 and 40 mg/kg for 8 consecutive days showed that the doses, respectively, inhibited the overexpression of the receptor for advanced glycation end products (RAGE), thereby preventing Aβ-induced neuronal toxicity [36]. In another study, continuous dietary supplementation with protocatechuic acid (Sigma Company, purity 99.5%) at doses of 0.5, 1, or 2 g for 8 weeks was evaluated in mice. Compared with the galactose-induced aging model group, supplementation with 1 or 2 g of protocatechuic acid reduced the generation and accumulation of methylglyoxal by inhibiting aldose reductase and activating glyoxalase I, thereby lowering the levels of advanced glycation end products (AGEs) [131]. In a galactose-induced aging model, intragastric administration of baicalein (Shanghai Aladdin Biochemical Technology Co., Ltd., with a purity of 98.5%) at 50 mg/kg daily for 8 consecutive weeks significantly reduced AGE accumulation, inhibited RAGE expression, and exerted anti-aging effects [132]. Furthermore, in naturally aging rats, continuous administration of kaempferol (source and purity unspecified) at doses of 2 and 4 mg/kg for 10 days showed that the 4 mg/kg dose inhibited RAGE expression and the accumulation of AGEs [133]. Additionally, chrysin (Sigma, purity ≥ 96.5%, concentration unknown) [134] and myricetin (Yuanye, Shanghai, China, purity unknown, 40, 60, 80 μmol/L) [135] exhibited potent anti-glycation activity in the human serum albumin and fructose-glycated bovine serum albumin glycation reaction systems, respectively.

### 4.9. Other Mechanisms

In addition, AOF and its bioactive metabolites can effectively prevent and control AD by counteracting neuroexcitotoxicity, addressing metabolic disorders, targeting neuroinflammatory responses, and activating endogenous antioxidant defense systems. Specifically, 25 mg/kg and 50 mg/kg of baicalein (Sigma, purity unspecified, a bioactive metabolite of AOF) were orally administered to APP/PS1 mice for 14 consecutive days, and the cognitive function of AD mice improved through the influence of the anti-glutamic acid pathway and mitigation of neuronal damage induced by excitotoxicity [136]. Moreover, consecutive oral administration of 360 mg/kg AOF total extract (Tongrentang Drug Co., Ltd., Henan, China) over 3 weeks alleviated memory and learning deficits in AD rats by regulating amino acid, lipid, and energy metabolism [3]. The purified polysaccharide components (2.5, 5, 10 μM) from AOF exert neuroprotective effects by inhibiting the TLR4/MyD88/NF-κB pathway to reduce NO, IL-1β, and TNF-α production in LPS-induced BV2 microglia in a concentration-dependent manner, while alleviating ROS accumulation and inhibiting neuronal apoptosis in SH-SY5Y cells [137]. In conclusion, the aforementioned studies, especially the in vivo studies, indicate that bioactive metabolites in AOF exert neuroprotective effects through multi-target synergistic mechanisms. For instance, in a mouse model of Aβ-induced memory impairment, tectochrysin exhibited significant neuroprotective effects by inhibiting oxidative stress, reducing cholinesterase activity, and decreasing Aβ1–42 accumulation in the brain [32]. Nootkatone exhibited dual antioxidant and AChE-inhibitory activities and inhibited abnormal Aβ deposition, thereby effectively improving Aβ-induced memory dysfunction [75]. Moreover, most AOF metabolites exhibit multiple pharmacological activities, including antioxidant, anti-inflammatory, and Aβ-deposition effects, demonstrating the overall regulatory characteristics of AOF in the intervention of neurodegenerative diseases through multiple components, targets, and pathways.

### 4.10. Aβ/Tau Hub and AOF Multi-Target Amelioration

The aggregation of Aβ and p-Tau, which leads to neurofibrillary tangles, is a significant pathological driving factor of AD. This section discusses many regulatory mechanisms in peripheral tissues and the central nervous system that can induce and exacerbate the accumulation of Aβ and/or Tau [138]. The dysregulation of the microbiota in peripheral tissues [139], metabolic disorders in the body [140], and epigenetic changes [141] can all trigger neuroinflammation, activate β-site amyloid precursor protein cleaving enzyme 1 and amyloid precursor protein processing, and increase the excessive production of Aβ and the phosphorylation of Tau in the brain [142]. In the central nervous system, elevated metal ions (Cu^2+^, Zn^2+^, Fe^2+^) in the brain can activate oxidative stress, impair autophagy, induce synaptic dysfunction, and exacerbate excessive Aβ and Tau aggregation [113]. Furthermore, abnormal expression of AChE may affect APP processing and Aβ production indirectly by regulating the level of the γ-secretase catalytic subunit PS1 [143].

Meanwhile, the exosomes transport pathological proteins to adjacent brain regions [120,144], thereby creating a self-amplifying process of Aβ and Tau deposition in the brain. Furthermore, the excessive accumulation of Aβ and/or Tau in the brain can trigger multi-level cascading damage [145] and directly activate microglia to transform into the pro-inflammatory M1 phenotype, releasing inflammatory factors such as IL-1B and TNF-a, activating the nuclear factor kappa-B pathway, and inhibiting neuronal autophagy, thereby blocking the clearance of Aβ and Tau themselves [146]. Excessive aggregation of Aβ and/or Tau can also induce excessive ROS production in mitochondria and upregulate the expression of β-site amyloid precursor protein cleaving enzyme 1, promoting the generation of more Aβ. This creates a self-reinforcing cycle of “Aβ/Tau neuroinflammation/oxidative stress/more Aβ/Tau” [147].

AOF extract exhibits strengths in AD-ameliorating potential, as its diverse active metabolites can act on several pathological mechanisms in parallel. For example, yakuchinone A and kaempferol can enhance autophagy [38,110]. Chrysin promotes the M2 polarization of microglia, enhancing Aβ phagocytosis and clearance [97]. Baicalein can reduce iron accumulation, thereby inhibiting oxidative stress in the brain and reducing exosome production [115]. Meanwhile, myricetin can also exert neuroprotective effects by regulating epigenetic modifications [126]. Furthermore, AOF can regulate the structure of the intestinal flora and its metabolites, thereby reducing the input of peripheral systemic inflammation to the central nervous system [100]. Therefore, the multi-target AD-ameliorating advantage of AOF is not the independent pharmacological effect of a single pathway, but the result of the coupling and coordinated interaction of multiple pathways, forming a multi-node and multi-level collaborative protection network.

## 5. Research Progress on AOF and AD

### 5.1. Approved Available Products

The mainstream drugs used for AD treatment primarily comprise cholinesterase inhibitors, memantine, brexpiprazole, and recently approved monoclonal antibodies, such as aducanumab, lecanemab, donanemab and benzgalantamine. Regarding the approved drugs for AOF, comprehensive retrievals were performed across multiple authoritative regulatory databases and published literature resources, including the databases of the United States Food and Drug Administration, European Food Safety Authority, European Chemicals Agency, Japan Pharmaceuticals and Medical Devices Agency, South Korea Ministry of Food and Drug Safety, and China National Medical Products Administration, among others. Approved AOF formulations for neurological diseases are shown in Table 3. A search performed across the literature and authoritative regulatory databases indicates that most studies on AOF-related drugs for neurological diseases originate in China, with limited reports coming from other countries. Moreover, existing AOF-related research mostly focuses on its use in combination within traditional Chinese medicine compound formulas, with few studies examining its application as a single herb. Prominent examples include Jian Nao Capsules, Jian Nao Tablets, Ning Xin Yi Zhi Capsules, Ning Xin Yi Zhi Oral Liquid, and Tonifying Brain Pills, which mainly contain AOF and are used to treat the neurological decline and memory loss associated with AD (Table 3). The compound compatibility exerts synergistic neuroprotective effects and avoids the stability defects of the AOF monomer, but it blurs its independent pharmacological contribution and impedes mechanism clarification.

### 5.2. Clinical Research

Advances have been made in investigating the effects of AOF and its individual bioactive metabolites on AD (Table 4). (1) Quercetin is essentially the only bioactive metabolite of AOF with potential pharmacological effects on AD that has undergone clinical investigation in humans [148]. Unfortunately, quercetin is not a major constituent of AOF due to its low content. Meanwhile, AOF cannot be used as a primary source for quercetin extraction. (2) Several compound formulations containing AOF have demonstrated clinical effects in randomized controlled trials, with significant improvements in cognitive function [149,150,151,152]. The specific details of these trials are provided in Table 4. (3) Certain AOF mixtures have been reported to ameliorate AD in phase I and phase II trials. For example, a decoction prepared from AOF, *Codonopsis pilosula* (Franch.) Nannf. (*Campanulaceae*; *Codonopsis pilosula* radix) and *Atractylodes macrocephala* Koidz. (*Asteraceae*; *Atractylodes macrocephala* rhizoma) has entered a phase I trial. Moreover, a granule formulation containing AOF, *Polygonum cuspidatum* Siebold & Zucc. (*Polygonaceae; Reynoutria japonica* radix et rhizoma), and *Salvia miltiorrhiza* Bunge (*Lamiaceae*; *Salvia miltiorrhiza* radix et rhizoma) has been approved for exploratory trials for the treatment of AD. (4) Additionally, a phase II clinical trial of a capsule primarily composed of AOF and *Polygonum multiflorum* Thunb. (*Polygonaceae*; *Polygonum multiflorum* radix) for the treatment of vascular dementia is currently in the subject recruitment stage. Several compound formulations containing AOF have demonstrated clinical effects in randomized controlled trials, showing significant improvements in cognitive function.

The clinical evidence summarized in Table 4 warrants cautious interpretation, as the vast majority of positive outcomes were derived from polyherbal decoctions or capsules in which AOF was combined with other medicinal plants. Consequently, the specific contribution of AOF to the overall alleviative efficacy cannot be isolated from the synergistic or confounding effects of the concomitant herbs. Regarding AOF as a standalone agent, no independent clinical trial has yet been conducted using its purified single extract in patients with AD, representing a noticeable knowledge gap in the current translational pipeline. As for individual constituents, although quercetin—a flavonoid present in AOF—has demonstrated neuroprotective properties in preclinical settings, it is neither unique to AOF nor representative of the full phytochemical complexity of the fruit. Therefore, the current clinical data should be regarded as supportive, albeit non-specific, background evidence, and rigorous Phase I/II trials employing standardized AOF monotherapy are urgently warranted to definitively ascertain its clinical efficacy.

### 5.3. Preclinical Research

The regulatory effects of AOF crude extracts and the metabolites directly extracted from the AOF raw material in AD animal studies are summarized and listed in Table 5. In vivo experiments have confirmed that AOF extracts (using ethanol, chloroform, petroleum ether, or n-butanol) exert neuroprotective effects by inhibiting AChE activity, regulating metabolic disorders, producing anti-inflammatory effects, and reducing Aβ levels [3,21,73,82]. Moreover, studies on metabolites directly extracted from AOF raw materials in AD animal models are relatively scarce and limited to nootkatone [75]. Additionally, metabolites directly extracted from AOF raw materials, including izalpinin [38], 2β-hydroxy-δ-cadinol [48], yakuchinone A [38], and yakuchinester A [38], have been verified to exert AD-ameliorating effects in AD cell models. The doses, intervention protocols, and corresponding effects are presented in Table 2 (effects of AOF bioactive metabolites on AD in cell studies) in Section 3.

### 5.4. Critical Appraisal: Discrepancies Between Preclinical and Clinical Realities

In vitro experiments were conducted in isolated and simplified systems, which have fundamental differences from in vivo experiments in terms of system complexity and the dose–concentration relationship. Firstly, cell experiments mostly use a single cell type, while the “multi-target” characteristics of the raw materials of Chinese medicine AOF or its extract often need to be fully demonstrated in a complex tissue microenvironment. Secondly, the metabolites directly extracted from AOF raw materials, including izalpinin, 2β-hydroxy-δ-cadinol, yakuchinone A, and yakuchinester A, have achieved effective concentrations at the cellular level; however, systematic pharmacokinetic data are still lacking to support whether these metabolites can reach the same exposure level in the target organs after oral absorption, distribution, metabolism, and excretion. Therefore, the characteristic metabolites listed in Table 2 still need to be researched further with respect to their efficacy through standardized in vivo studies.

Current clinical studies and approved available products of AOF for AD are limited to herbal formulations combining AOF extracts with other traditional Chinese medicine components (Table 3 and Table 4). AOF crude extracts and nootkatone (a metabolite directly extracted from AOF raw materials) have demonstrated AD-ameliorating potential in animal experiments (Table 5); however, none of these substances has yet been developed as a standalone drug for AD treatment due to considerable translational challenges from animal models to clinical use. The core of these challenges lies in the gap between the properties of the drugs themselves, the limitations of animal models, and the complexity of clinical research. First, many natural extracts and metabolites suffer from poor absorption, rapid metabolism and limited brain penetration following oral or injection intervention, resulting in an inability to reach the effective concentrations in the human body as observed in animal experiments. For example, nootkatone is rapidly absorbed after oral administration and reaches peak concentration within approximately 8 min; however, its mean residence time is only about 0.5 h [153]. The oral bioavailability is extremely low (1.51%), indicating remarkable first-pass elimination [153]. Its plasma concentration decreases to approximately 10 ng/mL at 1 h post-dose administration and further drops to around 2 ng/mL at 2 h, demonstrating rapid in vivo clearance [153]. Moreover, the extremely low oral bioavailability is a common problem for many flavonoid compounds [154]. Chrysin, a characteristic bioactive metabolite of flavonoids in AOF extracts, has poor water solubility and oral absorption [155]. Second, drugs must pass through the blood–brain barrier to exert their effects on the central nervous system. This is a significant challenge for many compounds. While a study predicted to passive permeation through the blood–brain barrier, the penetration capacity of chrysin is reportedly limited [156]. Tectochrysin, a characteristic bioactive metabolite in AOF extract, may exhibit weaker blood–brain barrier penetration than chrysin [157]. Third, the problems of dose conversion and safety assessment still exist. How to convert the effective dose (mg/kg) from animal experiments to an equivalent dose for humans is a controversial scientific issue. Patients with AD often need long-term medication. Animal studies generally have short experimental durations, making it difficult to detect the chronic toxicity of the test agent; accordingly, data on long-term safety remain scarce. Hence, further research is needed on the properties, mechanisms, and clinical translation of AOF extracts and their active metabolites. For example, future research may explore delivery strategies such as nano-formulations to improve the bioavailability and blood–brain barrier penetration of nootkatone, thereby enhancing its effects in the central nervous system.

## 6. AI-Powered Drug Discovery and Future AOF Research Directions

AI has demonstrated significant value in analyzing complex biological systems, identifying disease biomarkers and potential drug targets, simulating drug–target interactions, and predicting the safety and pharmacological effects of candidate drugs [158]. Therefore, AI has potential applications across almost all stages of drug development. Although the application of AI in the development of drugs based on AOF is still in its early stages, it is expected to advance research on AOF extraction; screening; absorption, distribution, metabolism, excretion, and toxicity (ADMET) prediction; and regulatory mechanisms, as well as facilitate related clinical trials.

### 6.1. Optimization of the Extraction Processes Using AI

Volatile oils in AOF are typically extracted using steam distillation, whereas flavonoids, terpenoids, and other bioactive metabolites are commonly extracted using ethanol or other organic solvents. The selection of appropriate solvents and extraction processes is crucial for achieving efficient recovery of bioactive metabolites. AI technology has promoted a transition in natural-product extraction processes from empirical trial-and-error approaches to data-driven intelligent design. Its core lies in accurately mapping the complex “process–product” relationship through machine learning models and applying intelligent optimization algorithms to achieve goal-oriented global optimization. During ultrasonic extraction of total flavonoids from *Agrimonia pilosa* Ledeb. (*Rosaceae*; *Agrimonia pilosa* herba), yield was used as the sole optimization objective. A tandem strategy combining response surface methodology, artificial neural networks, and genetic algorithms was adopted. This strategy enabled efficient global searches across a high-dimensional neural-network-constructed response surface, the direct identification of conditions associated with maximal yield, and levels of accuracy and efficiency unattainable using traditional methods [159]. In extraction processes involving traditional Chinese medicine compound formulations, radial basis function neural networks combined with nondominated sorting genetic algorithm II have been used to simultaneously maximize polysaccharide and secondary metabolite yields while minimizing energy consumption. This approach has generated a series of non-dominated solutions (Pareto solution sets) that provide a scientific basis for process prediction, optimization, and decision-making [160]. In AOF, different bioactive metabolites such as terpenoids, flavonoids, and volatile oils respond differently to extraction parameters, and complex synergistic or antagonistic interactions often exist among their solubility characteristics. In the future, extraction processes targeting single metabolites (such as total flavonoids) may be developed to adopt tandem optimization frameworks integrating response surface methodology, artificial neural networks, and genetic algorithms to achieve global optimization of the process parameters and thus substantially improve extraction efficiency. When the simultaneous optimization of multiple bioactive metabolites and multiple objectives (such as energy consumption and cost) is required, multi-objective evolutionary algorithms coupled with artificial neural networks, such as nondominated sorting genetic algorithm II-based frameworks, may be applied to generate Pareto-optimal solution sets. These approaches could provide a scientific and quantifiable basis for balancing trade-offs among competing objectives. Overall, AI-based process optimization technologies provide advanced and highly promising tools for multicomponent collaborative extraction and process design in the complex traditional Chinese medicine system of AOF.

### 6.2. The Use of AI in Drug Screening

Potential active metabolites in AOF with potential effects on AD have been screened using computational approaches such as network pharmacology and molecular docking. Using these strategies, several key metabolites that likely regulate pathogenic pathways associated with neurodegenerative diseases have been preliminarily predicted, including oxyhylladiketone, oxyphyllacinol, butyl-β-D-fructopyranoside, and dibutyl phthalate [71]. However, the application of network pharmacology in ethnopharmacology is constrained by limited predictive validity and insufficient biological relevance due to multi-layered homogeneity across distinct research objects [72]. Moreover, the predictive potential of these approaches is constrained by the availability of information in existing databases, thereby limiting the discovery of novel bioactive metabolites. Recently, Zhou et al. developed a transformer-based chemical language model called SELFormer, which utilizes extensive libraries of natural metabolites to directly predict molecules with potentially high activity on multiple core AD targets. Subsequent uniform manifold approximation and projection clustering and molecular docking were performed, further narrowing the candidates to 17 molecules with potential alleviative value [161]. Compared with traditional network pharmacology approaches, this strategy is not restricted by the limited component lists available in existing databases and therefore enables a transition from “explaining the known” to “discovering the unknown” [161]. In the future, pretrained large-scale chemical models may be used to directly evaluate all possible chemical structures present in AOF, including many unrecorded trace metabolites and derivatives. This approach may overcome current material limitations and enable the accurate prediction and identification of novel lead metabolites with strong bioactive targets associated with neurodegenerative diseases. In the future, such advances will not only provide a more comprehensive understanding of the pharmacological basis of AOF but also identify candidate metabolites with potential for entry into the drug development pipeline.

Furthermore, in a fibrosis-focused study, a large volume of multi-omics data was uploaded to the PandaOmics platform, which leverages natural language processing and deep neural network architectures to precisely identify targets highly correlated with the pathological mechanisms of fibrosis and possessing favorable “druggability” from tens of thousands of potential candidates. Subsequently, based on the identified targets, Chemistry 42 was employed to rationally design molecules with low synthetic accessibility hurdles and high “druggability” according to ideal drug design principles. These generated molecules served as preclinical candidates and were successfully applied in both preclinical studies and clinical models [162]. Currently, the bioavailability and activity of bioactive metabolites extracted from AOF exhibit certain limitations. Drawing on the concept of AI-driven drug design, generative AI platforms such as Chemistry 42 may be used in the future to optimize the core structures of bioactive AOF metabolites and design derivatives with greater activity and lower toxicity. This would extend current research from the characterization of natural products to structural optimization and innovative drug discovery.

### 6.3. ADMET Prediction Using AI

Drug ADMET is a crucial evaluation indicator and is used for pharmacokinetic and safety assessments of drugs in vivo. Current pharmacokinetic assessments of AOF’s active ingredients mainly rely on the “Lipinski rules + oral bioavailability” evaluation method [163]. This method essentially serves as an auxiliary preliminary screening tool, relying mainly on basic physicochemical indicators such as molecular weight and lipid solubility. It is difficult to accurately predict whether a drug will retain its activity after crossing the blood–brain barrier using this method. Additionally, it neglects metabolic transformation in the body and potential toxicity, resulting in deviations between the predicted results and the actual drug activity, alongside insufficient accuracy. In contrast, an intelligent prediction system based on large language models pretrained by treating Simplified Molecular Input Line Entry System (SMILES) sequences as a chemical language enables the model to master the basic grammatical rules of chemical structures. It then performs multi-task fine-tuning for the core drug ability attributes of AD (e.g., blood–brain barrier permeability) to construct an integrated prediction framework. It can automatically extract high-order semantic features from molecular sequences, achieving a combined, precise assessment of multiple dimensions of attributes, such as absorption, distribution, metabolism, excretion, and toxicity [164]. These large language models could be leveraged in future efforts to discover AD-improving drugs from AOF. For instance, by hypothetically inputting the SMILES sequence of the identified bioactive metabolites from AOF into a pre-trained model (trained on chemical big data and fine-tuned for brain-targeted ADMET properties), detailed predictions could be obtained for each compound. Such an approach would enable a comprehensive, quantitative assessment of the medicinal potential of each component. It would potentially overcome the limitations of the traditional “Lipinski rules + oral bioavailability” methods. This would apply particularly to brain-targeted compatibility and toxicity warnings. However, this strategy has not yet been empirically applied to AOF in the literature.

Furthermore, AI has been used to directly model molecular structures as graph data by constructing pharmacokinetic prediction frameworks based on graph neural networks and ensemble learning. Using graph neural networks, the topological features of atoms and chemical bonds can be automatically extracted, whereas stacked ensemble strategies integrate the strengths of multiple models to achieve the high-precision end-to-end prediction of ADMET properties [165]. This approach overcomes the reliance on manually designed descriptors and enables the simultaneous quantitative evaluation of multiple dimensions, including absorption, distribution, metabolism, and toxicity. It exhibits stronger predictive capability for complex properties, such as brain targeting. In the future, bioactive metabolites of AOF may be converted into molecular graphs and input into this model. This would enable a precise and comprehensive assessment of blood–brain barrier permeability, metabolic stability, and cardiac toxicity for each component. This strategy would also facilitate the systematic ranking and optimization of pharmacologically bioactive metabolites within the complex AOF system and provide important support for the targeted development of neuroprotective lead metabolites.

### 6.4. Exploration of Regulatory Mechanisms Using AI

Research on the effects of AOF on AD has shifted from single-pathway verification to the systematic exploration of multi-target networks. Current evidence indicates that bioactive metabolites in AOF can inhibit Aβ deposition, reduce p-tau levels, and exert anti-inflammatory and antioxidant effects. However, these studies have mainly demonstrated the effects of AOF metabolites on terminal pathological markers, such as Aβ and tau. The mechanisms underlying the synergistic interactions among different active components remain unclear. In addition, the core hub targets co-regulated during the progression of AD remain unidentified at the systems level through approaches such as protein interaction network analysis and multi-omics integration. Based on AI technology, a systematic framework integrating computational prediction, network pharmacology, and experimental validation was established to facilitate multi-target research on AD using a dataset containing neuroprotective activity data for 253 molecules. First, machine learning models were used to preliminarily screen the compound library. Subsequently, a protein interaction network associated with AD was constructed to identify key upstream targets involved in AD pathology, such as mTOR and B-cell lymphoma 2. Finally, molecular docking and molecular dynamics simulations were performed to verify the stable binding of the selected natural compounds to mTOR and B-cell lymphoma 2, thereby enabling the prediction of systems-level synergistic effects involving the promotion of mTOR and inhibition of neuronal apoptosis. This strategy may provide an integrated multi-target, multi-pathway ameliorative approach for the complex pathological networks underlying AD [166].

Furthermore, the Priority Index Solution for AD (PI4AD) computational framework has been used to integrate multi-omics data with artificial neural networks to reconstruct the pathological network of AD [167]. For instance, through prioritization analysis, the PI4AD framework identified the renin–angiotensin–aldosterone system signaling pathway, in which renin–angiotensin–aldosterone system proteins function as molecular switches, a central hub linking synaptic dysfunction and neuroinflammation. This finding highlights the importance of multi-target combinational interventions directed at this pathway [167]. The hub role of the renin–angiotensin–aldosterone system signaling pathway identified using the PI4AD framework provides a novel computational perspective for uncovering the intrinsic relationships between regulatory mechanisms and target interaction networks. As a natural product with multi-target regulatory potential, bioactive metabolites in AOF have been experimentally shown to intervene in AD pathology through multiple mechanisms, including the inhibition of neuronal apoptosis, regulation of neuroinflammation, and promotion of autophagy. If this systematic model or the PI4AD computational framework is adopted in future studies, additional regulatory mechanisms of AOF and their intrinsic relationships may be further explored.

### 6.5. The Use of AI at the Clinical Trial Stage

The emergence of real-world data and digital twin technology has brought about new opportunities in the field of clinical trials [168]. CD-Tro is a clinical language model developed through fine-tuning of the GatorTron large language model, and it can be used to efficiently mine unstructured text from electronic health records, enabling the early prediction of mild cognitive impairment up to 4 years before a clinical diagnosis. The model demonstrated near-perfect discrimination and extremely high sensitivity in an independent test set, while SHapley Additive exPlanations (SHAP)-based interpretability analysis enhanced its clinical credibility. It can provide direct technical support for the application of natural language processing technology to accurately identify high-risk populations using real-world data. Accordingly, natural language processing may be used to automatically and rapidly identify patients meeting clinical trial criteria from millions of medical records, thereby generating precise lists of potential participants [169]. Applying this approach in future studies may help address the inefficiency of traditional subject screening for natural products such as AOF, which often results in delayed trial initiation and elevated costs.

Moreover, in the Digital Twin Trial methodology proposed by Andrews et al., the AD Neuroimaging Initiative database was used to construct a simulated 18-month improvement trial scenario [170]. By matching each participant with a “digital twin” possessing similar baseline characteristics and using the historical cognitive decline trajectory to adjust observed outcomes, within-group variance was significantly reduced. The results showed that the Digital Twin Trial approach reduced sample size requirements by approximately 9.5–19% compared with those of traditional randomized controlled trials [170]. If this methodology is adopted in future research, it may alleviate recruitment pressure in clinical trials of natural products such as AOF and improve the ethical feasibility of such studies.

In summary, AI technology, through its robust data processing and intelligent analytical capabilities, has become deeply integrated into multiple stages of drug development. It is currently transforming traditional drug screening paradigms and process-optimization strategies. If these technologies are applied to AOF in future work, they will lay the groundwork for intelligent, precision-guided AOF drug development. Thus, AI technology represents a critical driving force for innovation in the pharmaceutical field. However, many AI models remain primarily data-driven, and their effectiveness in drug development is constrained by the limited availability of high-quality datasets. Improving the accuracy and generalizability of these models remains a major challenge. Moreover, AI-designed drugs and AI-based property predictions still require experimental validation through wet lab studies.

## 7. Conclusions and Future Perspectives

The Chinese medicinal and edible herb AOF contains flavonoids, terpenoids, sesquiterpenoids, diarylheptanes, and volatile oils. Because of these bioactive metabolites, AOF has been shown to exert unique effects that significantly enhance cognitive function and provide neuroprotection [171]. The AD-ameliorating effects, absorption, and toxicity of AOF extracts have been extensively reported. Among AOF metabolites, only a limited number have been investigated in animal models of AD, including myricetin, baicalein, pinocembrin, rhamnocitrin, galangin, linalool, α-terpineol, eucalyptol, nootkatone, and β-caryophyllene. Quercetin has also been studied in humans. Overall, substantial opportunities remain for further investigations into metabolites from AOF and their potential application as alleviative agents. Furthermore, the multi-target mechanisms by which AOF extracts and their monomeric components improve AD have been investigated, particularly for emerging pathways involving glial cell function, the gut–brain axis, brain metal ion homeostasis, and epigenetic regulation (e.g., AGEs-related mechanisms). These studies have elucidated the mechanisms by which AOF and its bioactive metabolites regulate pathological pathways and intervene in the progression of AD, providing molecular-level explanations for their alleviative effects. In summary, AOF exhibits multi-component and multi-target properties and has the potential to serve as a novel neuroprotective agent, representing a promising option for ameliorating neurodegenerative diseases.

Although AOF and its metabolites possess remarkable research significance and enormous developmental potential, their translational development and industrial application are constrained by several challenges. First, only a small number of AOF metabolites have been verified to exert AD-ameliorating and neuroprotective effects in vivo. However, many metabolites from AOF, including terpenoids, flavonoids, diphenylheptanes, and sterols, exhibit diverse biological activities, including anticancer, anti-inflammatory, antioxidant, and gastrointestinal protective effects. Several bioactive metabolites, including sesquiterpenoids, terpenoids, phenolic acids, and furans, are present in AOF, and they are promising candidates for further research on AD. Second, the officially recognized bioactive components of AOF in the Chinese Pharmacopoeia are currently limited to volatile oils. However, as additional active constituents (e.g., flavonoids, terpenes, and sesquiterpenes) continue to be identified, both in vivo effect evaluations and quality control standards for new components must be updated accordingly. In recent years, numerous studies and patents have focused on novel separation techniques for rare terpenoids and sesquiterpenoids, as well as identification methods for new monomer components, with relevant intellectual property protection being progressively implemented. Third, further exploration of effective strategies for targeting active monomers in AOF for AD improvement represents an important research direction. The use of AI and related technologies to screen and design highly selective active compounds with minimal side effects may not only improve targeting accuracy and alleviative precision but also facilitate breakthroughs in improving AD.

Furthermore, the clinical translation of AOF-related drugs requires a balanced consideration of aspects such as safety, metabolism, and drug interactions. The safe dosage of the unprocessed herbal material of AOF (crude AOF) is 3–9 g/day. An acute toxicity study has shown that no obvious toxicity was observed after a single administration of 1000 mg/kg of AOF extract [172]. In terms of metabolism, pharmacokinetic studies have identified 27 metabolites derived from five terpenoid compounds in the plasma, urine, and feces of rats following oral administration of AOF extract [173]; however, these findings are limited to rodents, and human pharmacokinetic studies on the administration of AOF extract alone are currently unavailable. Regarding drug toxicity, AOF comes from plants of the Curcuma genus, and these plants may weaken the efficacy of histamine H_2_ receptor antagonists [174]. As a component of traditional Chinese medicine, crude AOF has no definite incompatibility contraindications; nevertheless, special populations such as pregnant women and individuals with allergic diathesis should exercise caution when taking preparations containing AOF. In addition to the AOF extract itself, the application of AOF metabolites also requires paying special attention to toxicity studies. In summary, the existing evidence supports the relative safety of AOF at the traditional dose, but the lack of long-term toxicological data, incomplete human pharmacokinetic characteristics, and unclear drug interaction spectrum constitute key knowledge gaps for its clinical application in AD treatments.

Additionally, pharmacokinetic relevance links in vitro efficacy to in vivo brain exposure. Qualitative evidence confirms that certain AOF metabolites—such as protocatechuic acid, chrysin, nootkatone, and tectochrysin [157]—can penetrate the blood–brain barrier, and 34 compounds have been detected in mouse brain tissue after oral administration of AOF extract [23]. These results fail to address the effective pharmacologically relevant concentrations of these drugs reaching the brain. However, this critical factor for the translational validity of preclinical data remains largely unaddressed in the current literature. Most existing studies only document direct alterations in behavioral readouts, Aβ, and Tau following drug intervention, and consequently, conclusions are drawn regarding the AD-ameliorating efficacy of tested agents, while neglecting assessments of brain-penetrating drug dosages. Future investigations could systematically quantify the dynamic drug-free concentrations in the brain and their corresponding pharmacokinetic profiles following administration, thus furnishing direct quantitative pharmacologic evidence for the exposure response relationship.

## Figures and Tables

**Table 1:** Effects of AOF bioactive metabolites on AD in animal studies.

Category	Matter (Source and Purity)	Model, Doses and Intervention	Effective Doses and AD-Ameliorating Mechanisms	Ref.
Flavonoids	Chrysin (MedChemExpress LLC., Monmouth Junction, NJ, USA, purity unspecified)	APP/PS1 mice; 25 mg/kg chrysin, intragastric administration for 180 days	25 mg/kg; inhibition of Aβ and enhancement of Aβ clearance	[30]
	Daucosterol palmitate (extract from AOF, purity > 98%)	Intraventricular injection of 10 μL Aβ1–42Aβ1–42 in Wistar rats; 24 mg/kg daucosterol palmitate, gavage, 14 days	24 mg/kg; antioxidation	[31]
	Tectochrysin (Jiangsu Jurong Pharmaceutical Co., Changzhou, China, purity > 99%)	Intraventricular injection of 3 µL Aβ1–42Aβ1–42 in Kunming mice; 14, 140 µg/d tectochrysin, intraventricular injection, 5 days	14, 140 µg/d; inhibition of Aβ accumulation	[32]
	Kaempferol (extract from *Mespilus germanica*, purity unknown)	10 μL of ICV injection of streptozotocin in Wistar rats; 10 mg/kg kaempferol, intraperitoneal injection, 21 days	10 mg/kg; antioxidation	[33]
	Myricetin (Sigma, St. Louis, MO, USA, purity ≥97%)	Intraperitoneal injection of 2.8 mg/kg trimethyltin chloride; 25 mg/kg myricetin, oral administration, 21 days	25 mg/kg; relief of endoplasmic reticulum stress	[34]
	Baicalein (Chemical Centre of Mumbai, Mumbai, India, purity 98%)	Intraventricular injection of 400 pmol Aβ1–42 in albino Wistar rats; 5, 10 mg/kg, oral administration, 21 days	5, 10 mg/kg; anti-inflammation	[35]
	Pinocembrin (Department of Medical Synthetic Chemistry, Institute of Materia Medica, Beijing, China, purity > 98%)	Intraventricular injection of 10 nmol Aβ25–35 in Kunming mice, 10, 20 mg/kg pinocembrin, oral gavage, 8 days	10, 20 mg/kg; inhibition of the overexpression of the receptor for advanced glycation end products	[36]
Terpenoids	Eucalyptol (source and purity unknown)	Intraventricular injection of 480 µg advanced glycation end product in SD mice; 50, 100, 150 mg/kg eucalyptol, gavage, 28 days	50, 100, 150 mg/kg; reduction in Aβ production	[41]
	Linalool (source and purity unknown)	Intraventricular injection of 0.5 mM Aβ1–42 in SD rats + 50, 100 mg/kg, intraperitoneal injection, 21 days	50, 100 mg/kg; anti-inflammation and antioxidation	[42]
	α-terpineol (Sigma, purity unknown)	Intraventricular injection of 2 µL Aβ1–42 in Wistar rats, 100 mg/kg α-terpineol, intraperitoneal injection, 7 days	100 mg/kg; reduction in amyloid plaques	[43]
	β-pinene (Sigma, purity unknown)	Intraventricular injection of 10 mg/kg streptozotocin; 50, 100, 200 mg/kg, oral gavage, 21 days	100, 200 mg/kg; antioxidation, anticholinesterase activity, and mitochondrial protection	[44]
	β-sitosterol (Sigma, purity unknown)	APP/PS1 mice; 20 mg/kg β-sitosterol, gavage, 28 days	20 mg/kg; reduction in Aβ deposition	[45]
Sesquiterpenoids	Nootkatone (Sigma, purity > 97%)	Intraventricular injection of 40 µg LPS in C57BL/6 mice; 5, 10 mg/kg nootkatone, oral administration, 19 days	5, 10 mg/kg; anti-inflammation	[49]
	β-caryophyllene (Tokyo Chemical Industry (India) Pvt. Ltd., Hyderabad, India, purity unknown)	Intraventricular injection of 5 µL streptozotocin; 10, 20 mg/kg β-caryophyllene, intraperitoneal injection, 28 days	10, 20 mg/kg; modulation of BDNF signaling pathway, enhancement of synaptic plasticity, and alleviation of neuroinflammation	[50]
Volatile oils	P-cymene (Sigma, purity unknown)	Intraventricular injection of 2 µL Aβ1–42Aβ1–42 in Wistar rats; 50, 100 mg/kg p-cymene, intraperitoneal injection, 21 days	50, 100 mg/kg; enhancement of neurogenesis and reduction in amyloid plaques	[56]

**Table 2:** Effects of AOF bioactive metabolites on AD in cell studies.

Category	Matter (Source and Purity)	Model, Dosage and Intervention	Effective Doses and Effects	Ref.
Flavonoids	Izalpinin (extract from AOF)	1.0 mM Aβ co-treated with 0.2, 0.5 and 1.0 mM izalpinin, 8 h	0.2, 0.5 and 1.0 mM; reversal of Aβ-induced injury	[38]
Terpenoids	2β-hydroxy-δ-cadinol (extract from AOF)	20µM Aβ1–42 co-treated with 10, 20, 30 µM 2β-hydroxy-δ-cadinol, 24 h	10, 20, 30 µM; antioxidation	[48]
Diarylheptanoids	Yakuchinone A (extract from AOF)	1.0 mM Aβ co-treated with 0.25, 0.5 and 1.0 mM yakuchinone A, 8 h	0.25, 0.5 mM; inhibition of β-site amyloid precursor protein cleaving enzyme 1 expression and enhancement of lysosomal autophagy	[38]
	Yakuchinester A (extract from AOF)	1.0 mM Aβ co-treated with 0.25, 0.5 and 1.0 mM yakuchinester A, 8 h	0.2, 0.5 and 1.0 mM; reversal of Aβ-induced injury	[38]

**Table 3:** Approved AOF formulation for neurological diseases.

Formulated Preparation	Plant Compounds	Source and Approval Number	Dosage and Drug Indications
Jian nao capsules	*Angelica sinensis* (Oliv.) Diels (*Angelica*; *Angelica sinensis* radix), 33.3 g; *Bambusa textilis* McClure (*Poaceae*; *Bambusa textilis* concretio), 13.3 g; *Cistanche deserticola Ma* (*Orobanchaceae*; *Cistanche deserticola caulis*), 26 g; *Dioscorea opposita Thunb.* (*Dioscoreaceae*; *Dioscorea polystachya rhizoma*), 26.7 g; *Gastrodia elata* Blume (*Orchidaceae*; *Gastrodia elata* rhizoma), 6.7 g; *Schisandra chinensis* (Turcz.) Baill. (*Schisandraceae*; *Schisandra* chinensis fructus), 20 g; *Platycladus orientalis* (L.) Franco (*Cupressaceae*; *Platycladus* orientalis semen), 5.3 g; *Salvia miltiorrhiza* Bunge (*Lamiaceae*; *Salvia miltiorrhiza* radix et rhizoma), 6.7 g; *Alpinia oxyphylla* Miq. (*Zingiberaceae*; *Alpinia oxyphylla* fructus), 20 g; *Panax ginseng* C.A.Mey. (*Araliaceae*; *Panax ginseng* radix et rhizoma), 6.7 g; *Polygala tenuifolia* Willd. (*Polygalaceae*; *Polygala tenuifolia* radix), 13.3 g; *Chrysanthemum × morifolium* (Ramat.) Hemsl. (*Asteraceae*; *Chrysanthemum × morifolium* flos capituli), 6.7 g; *Anemone altaica* Fisch. ex C.A.Mey. (*Ranunculaceae*; *Anemone altaica* rhizoma), 13.3 g; *Ziziphus jujuba* Mill. (*Rhamnaceae*; *Ziziphus jujuba* Semen), 53.3 g; *Lycium barbarum* Lam. (*Solanaceae*; *Lycium barbarum* fructus), 26.7 g	National Medical Products Administration of China, Z37020790	0.6 g, 3 times daily; indicated for memory decline, mild cognitive impairment in the elderly, and similar conditions
Jian nao tablets	*Ziziphus jujuba* Mill. (*Rhamnaceae*; *Ziziphus jujuba* Semen), 40 g; *Angelica sinensis* (Oliv.) Diels (*Angelica*; *Angelica sinensis* radix), 25 g; *Dioscorea opposita* Thunb. (*Dioscoreaceae*; *Dioscorea polystachya* rhizoma), 20 g; *Schisandra chinensis* (Turcz.) Baill. (*Schisandraceae*; *Schisandra* chinensis fructus), 15 g; *Bambusa textilis* McClure (*Poaceae*; *Bambusa textilis* concretio), 10 g; *Cistanche deserticola* Ma (Orobanchaceae; *Cistanche deserticola* caulis), 20 g; *Gastrodia elata* Blume (*Orchidaceae*; *Gastrodia elata* rhizoma), 5 g; *Platycladus orientalis* (L.) Franco (*Cupressaceae*; *Platycladus* orientalis semen), 4 g; *Alpinia oxyphylla* Miq. (*Zingiberaceae*; *Alpinia oxyphylla* fructus), 15 g; *Polygala tenuifolia* Willd. (*Polygalaceae*; *Polygala tenuifolia* radix), 10 g; *Anemone altaica* Fisch. ex C.A.Mey. (*Ranunculaceae*; *Anemone altaica* rhizoma), 10 g; *Lycium barbarum* Lam. (*Solanaceae*; *Lycium barbarum* fructus), 20 g; *Salvia miltiorrhiza* Bunge (*Lamiaceae*; *Salvia miltiorrhiza* radix et rhizoma), 5 g; *Panax ginseng* C.A.Mey. (*Araliaceae*; *Panax ginseng* radix et rhizoma), 5 g	National Medical Products Administration of China, Z20090222	1 g, 3 times daily; indicated for overuse of the brain, memory decline, neurasthenia, and similar conditions
Ning xin yi zhi capsules	*Panax ginseng* C.A.Mey. (*Araliaceae*; *Panax ginseng* radix et rhizoma), 50 g; *Astragalus mongholicus* Bunge (*Fabaceae*; *Astragalus mongholicus* radix), 150 g; *Polygonatum odoratum* (Mill.) Druce (*Asparagaceae*; *Polygonatum* odoratum rhizoma), 50 g; *Acorus tatarinowii* Schott (*Araceae*; *Acorus tatarinowii* rhizoma), 50 g; *Alpinia oxyphylla* Miq. (*Zingiberaceae*; *Alpinia oxyphylla* fructus), 50 g; *Polygala tenuifolia* Willd. (*Polygalaceae*; *Polygala tenuifolia* radix), 50 g; *Schisandra chinensis* (Turcz.) Baill. (*Schisandraceae*; *Schisandra* chinensis fructus), 125 g; *Nardostachys jatamansi* (D.Don) DC. (*Caprifoliaceae*; *Nardostachys jatamansi* radix et rhizoma), 50 g	National Medical Products Administration of China, Z20090937	0.9 g, 3 times daily; indicated for senile dementia, memory decline, and similar conditions
Ning xin yi zhi oral liquid	*Panax ginseng* C.A.Mey. (*Araliaceae*; *Panax ginseng* radix et rhizoma), 50 g; *Astragalus mongholicus* Bunge (*Fabaceae*; *Astragalus mongholicus* radix), 200 g; *Polygonatum odoratum* (Mill.) Druce (*Asparagaceae*; *Polygonatum* odoratum rhizoma), 75 g; *Acorus tatarinowii* Schott (*Araceae*; *Acorus tatarinowii* rhizoma), 75 g; *Alpinia oxyphylla* Miq. (*Zingiberaceae*; *Alpinia oxyphylla* fructus), 50 g; *Polygala tenuifolia* Willd. (*Polygalaceae*; *Polygala tenuifolia* radix), 75 g; *Schisandra chinensis* (Turcz.) Baill. (*Schisandraceae*; *Schisandra* chinensis fructus), 150 g; *Nardostachys jatamansi* (D.Don) DC. (*Caprifoliaceae*; *Nardostachys jatamansi* radix et rhizoma), 75 g	National Medical Products Administration of China, B20020457	10 mL, twice daily; indicated for neurasthenia, memory impairment, and similar conditions
Tonifying brain pills	*Angelica sinensis* (Oliv.) Diels (*Angelica*; *Angelica sinensis* radix), 75 g; *Lycium barbarum* Lam. (*Solanaceae*; *Lycium barbarum* fructus), 60 g; *Ziziphus jujuba* Mill. (*Rhamnaceae*; *Ziziphus jujuba* Semen), 120 g; *Platycladus orientalis* (L.) Franco (*Cupressaceae*; *Platycladus* orientalis semen), 45 g; *Alpinia oxyphylla* Miq. (*Zingiberaceae*; *Alpinia oxyphylla* fructus), 45 g; *Polygala tenuifolia* Willd. (*Polygalaceae*; *Polygala tenuifolia* radix), 30 g; *Gastrodia elata* Blume (*Orchidaceae*; *Gastrodia elata* rhizoma), 30 g; *Acorus tatarinowii* Schott (*Araceae*; *Acorus tatarinowii* rhizoma), 30 g; *Cistanche deserticola* Ma (Orobanchaceae; *Cistanche deserticola* caulis), 60 g; *Bambusa textilis* McClure (*Poaceae*; *Bambusa textilis* concretio), 30 g; *Juglans regia* L. (*Juglandaceae*; *Juglans regia* semen), 60 g; *Schisandra chinensis* (Turcz.) Baill. (*Schisandraceae*; *Schisandra* chinensis fructus), 45 g	National Medical Products Administration of China, Z61020095	2–3 g, 2–3 times daily; indicated for memory impairment, forgetfulness, and similar conditions

**Table 4:** Clinical studies on AOF-containing compound formulations.

Matter	Plant Compounds	Patient and Intervention	Effect	Ref.
Wenpi Tongluo Kaiqiao Formula	*Astragalus mongholicus* Bunge (Fabaceae; Astragalus mongholicus radix), *Alpinia oxyphylla* Miq. (*Zingiberaceae*; *Alpinia oxyphylla* fructus), *Panax notoginseng* (Burkill) F.H.Chen (*Araliaceae*; *Panax notoginseng radix* et rhizoma), *Acorus calamus* L. (*Acoraceae*; *Acorus calamus* rhizoma), *Polygonum multiflorum* Thunb. (*Polygonaceae*; *Polygonum multiflorum* radix), *Gynostemma pentaphyllum* (Thunb.) Makino (*Cucurbitaceae*; *Gynostemma pentaphyllum* herba)	AD patients; 200 mL formula, orally, 4 weeks	Improve cognitive function	[149]
Chinese medicine prescription	*Alpinia oxyphylla* Miq. (*Zingiberaceae*; *Alpinia oxyphylla* fructus) *Nelumbo nucifera* Gaertn. (*Nelumbonaceae*; *Nelumbo nucifera* semen), *Dioscorea opposita* Thunb. (*Dioscoreaceae*; *Dioscorea polystachya* rhizoma)	Patients with mild cognitive impairment; 27 g formula, orally, 3 months	Improve cognitive function	[150]
Ding Zhi Yi Cong Fang	*Codonopsis pilosula* (Franch.) Nannf. (*Campanulaceae*; *Codonopsis pilosula* radix), *Acorus tatarinowii* Schott (*Araceae*; *Acorus tatarinowii* rhizoma), *Polygala tenuifolia* Willd. (*Polygalaceae*; *Polygala tenuifolia* radix), *Alpinia oxyphylla* Miq. (*Zingiberaceae*; *Alpinia oxyphylla* fructus), *Angelica sinensis* (Oliv.) Diels (*Angelica*; *Angelica sinensis* radix), *Rehmannia glutinosa* (Gaertn.) Libosch. ex DC. (*Orobanchaceae*; *Rehmannia glutinosa* radix), *Paeonia lactiflora* Pall. (*Paeoniaceae*; *Paeonia lactiflora* radix), *Lycium barbarum* Lam. (*Solanaceae*; *Lycium barbarum* fructus), *Ligusticum Chuanxiong* Hort. (*Apiaceae*; *Ligusticum chuanxiong* rhizoma), *Prunus persica* (L.) Batsch (*Rosaceae*; *Prunus persica* semen), *Carthamus tinctorius* L. (*Asteraceae*; *Carthamus tinctorius* flos)	Patients with dementia; one dose of decoction, orally, 60 days	Improve cognitive function	[151]
Tong Luo Yi Zhi Tang	*Codonopsis pilosula* (Franch.) Nannf. (*Campanulaceae; Codonopsis pilosula* radix), *Cuscuta chinensis* Lam. (*Convolvulaceae*; *Cuscuta chinensis* semen), *Epimedium brevicornu* Maxim. (*Berberidaceae; Epimedium brevicornu* folium), *Alpinia oxyphylla* Miq. (*Zingiberaceae*; *Alpinia oxyphylla* fructus)*, Codonopsis pilosula* (Franch.) Nannf. (*Campanulaceae*; *Codonopsis pilosula* radix et rhizoma), *Acorus tatarinowii* Schott (*Araceae; Acorus tatarinowii* rhizoma), *Polygala tenuifolia* Willd. (*Polygalaceae*; *Polygala tenuifolia* radix), *Bambusa textilis* McClure (*Poaceae*; *Bambusa textilis* concretio)*, Polygonum multiflorum* Thunb. (*Polygonaceae*; *Polygonum multiflorum* radix)	Patients with cognitive impairment; one dose of decoction, orally, 180 days	Improve cognitive impairment	[152]

**Table 5:** Studies on AOF crude extracts and metabolites directly extracted from AOF raw materials in AD animal models.

Category	Matter (Source and Purity)	Model, Doses and Intervention	Effective Doses and Effects	Ref.
Extract of AOF	Chloroform extract AOF (Shenyang Tongrentang Drug Co., Ltd.)	Intraventricular injection of 3 μL Aβ1–42 in ICR mice; 180, 360 mg/kg Chloroform extract AOF, intragastric injection, 20 days	180, 360 mg/kg; inhibition of AChE activity	[82]
	Chloroform extract, Petroleum ether extract and N-butanol extract AOF (Shenyang Tongrentang Drug Co., Ltd.)	Intraperitoneal injection of 0.25 mg/kg in ICR mice; 360 mg/kg Chloroform extract, Petroleum ether extract and n-butanol extract AOF, gavage, 14 days	360 mg/kg; inhibition of excessive activation of microglia	[21]
	N-butanol extract AOF (Shenyang Tongrentang Drug Co., Ltd.)	Intraventricular injection of 3 μL Aβ1–42 in ICR mice; 180, 360 mg/kg N-butanol extract AOF, gavage, 20 days	180, 360 mg/kg; reduction in the level of Aβ	[73]
	total extract AOF (Henan Tongrentang Drug Co., Ltd.)	Intraventricular injection of 10 μL Aβ1–42 in SD rats; 360 mg/kg/d total extract AOF, gavage, 21 days	360 mg/kg; regulation of amino acid, lipid and energy metabolism	[3]
Sesquiterpenoids	Nootkatone (extract from AOF, purity > 98%)	Intraventricular injection of 3 μL Aβ1–42; 0.02, 0.2 mg/kg nootkatone, intraventricular injection, 5 days	0.02, 0.2 mg/kg; anti-oxidative and anti-AChE activities	[75]

## Data Availability

No new data were created or analyzed in this study. Data sharing is not applicable to this article.

## Abbreviations

The following abbreviations are used in this manuscript:

AchE	Acetylcholinesterase
AD	Alzheimer’s disease
AGEs	Advanced glycation end products
AOF	*Alpiniae oxyphyllae* Fructus
Aβ	β-amyloid protein
DESs	Deep eutectic solvents
DPZ	Donepezil
LPS	Lipopolysaccharide
M1	Classically activated
M2	Alternatively activated
mTOR	Mammalian target of rapamycin
PAINS	Pan-assay interference compounds
PI4AD	Priority Index Solution for AD
P-tau	Phosphorylated tau protein
